# Adolescent neurodevelopment and psychopathology: The interplay between adversity exposure and genetic risk for accelerated brain ageing

**DOI:** 10.1016/j.dcn.2023.101229

**Published:** 2023-03-15

**Authors:** Raluca Petrican, Alex Fornito

**Affiliations:** a Institute of Population Health, Department of Psychology, University of Liverpool, Bedford Street South, Liverpool L69 7ZA, United Kingdom; b The Turner Institute for Brain and Mental Health, School of Psychological Sciences, and Monash Biomedical Imaging, Monash University, Melbourne, VIC, Australia

**Keywords:** Stress Susceptibility, Psychopathology, Major Depressive Disorder, Alzheimer’s Disease, Adolescent Development, Transcriptomics

## Abstract

In adulthood, stress exposure and genetic risk heighten psychological vulnerability by accelerating neurobiological senescence. To investigate whether molecular and brain network maturation processes play a similar role in adolescence, we analysed genetic, as well as longitudinal task neuroimaging (inhibitory control, incentive processing) and early life adversity (i.e., material deprivation, violence) data from the Adolescent Brain and Cognitive Development study (N = 980, age range: 9–13 years). Genetic risk was estimated separately for Major Depressive Disorder (MDD) and Alzheimer’s Disease (AD), two pathologies linked to stress exposure and allegedly sharing a causal connection (MDD-to-AD). Adversity and genetic risk for MDD/AD jointly predicted functional network segregation patterns suggestive of accelerated (GABA-linked) visual/attentional, but delayed (dopamine [D2]/glutamate [GLU5R]-linked) somatomotor/association system development. A positive relationship between brain maturation and psychopathology emerged only among the less vulnerable adolescents, thereby implying that normatively maladaptive neurodevelopmental alterations could foster adjustment among the more exposed and genetically more stress susceptible youths. Transcriptomic analyses suggested that sensitivity to stress may underpin the joint neurodevelopmental effect of adversity and genetic risk for MDD/AD, in line with the proposed role of negative emotionality as a precursor to AD, likely to account for the alleged causal impact of MDD on dementia onset.

Adolescence is a critical developmental phase, marked by a multitude of interdependent neurobiological and socio-environmental changes that are programmed to maximise adjustment to one’s milieu (Alvarez-Dominguez and Melton, 2022, Lopez et al., 2021). Importantly, this is also the life stage in which more than a third of all diagnosed mental disorders have their onset (Solmi et al., 2022), a finding that underscores the urgency in comprehensively characterising the determinants and markers of subsequent adaptation versus vulnerability (Akarca et al., 2021; Tozzi et al., 2020).

Accelerated brain ageing has been identified as a robust correlate of psychiatric and neurodegenerative risk across the life course (Kaufmann et al., 2019). Previous adult studies have provided compelling evidence that changes in the pace of brain ageing, which manifest as deviations from normative functional and structural neural connectivity, reflect interactions between genetic vulnerabilities and prior exposure to environmental stressors (for a review, see Goldsmith et al., 2022). Nonetheless, it is unclear whether brain maturation processes similarly mediate the joint impact of early life adversity and genetic risk on psychological functioning during the transition from childhood to adolescence. Addressing this question may help shed light on candidate neurodevelopmental mechanisms that could be targeted through early interventions, prior to or very early after clinical onset, which would more effectively alleviate maladaptive maturational trajectories.

To probe the developmental impact of early life adversity, we adopted a dimensional approach focused on exposure to deprivation (of material resources needed for optimal functioning) and threat/violence (i.e., family conflict, neighbourhood crime), both of which had been shown to impact brain maturation pace and psychopathology risk in adolescence (Colich et al., 2020; Cummings and Miller-Graf, 2015; Gard et al., 2022; Harold & Sellers, 2018; McLaughlin et al., 2020; Petrican et al., 2021).

To characterise the role of genetic vulnerability factors to psychological functioning in adolescence, we focused on Alzheimer’s Disease (AD) and Major Depressive Disorder (MDD), two conditions associated with accelerated brain ageing and increased susceptibility to environmental stressors (Gonneaud et al., 2021, Han et al., 2021, Kaufmann et al., 2019, Lutz et al., 2020, Millar et al., 2022). Both disorders have been linked to maturational processes, in particular, genes regulating lifespan neurodevelopment (Brouwer et al., 2022) and exposure to adversity in childhood/adolescence (Donley et al., 2018, Epperson et al., 2017, Ho and King, 2021, Norton et al., 2011, Radford et al., 2017, Tani et al., 2020). Recent genetic correlation- and Mendelian randomisation-based evidence suggests that MDD may be causally predictive of subsequent AD onset (but not vice-versa; Harerimana et al., 2022). This conjecture is particularly consequential given that onset of the first depressive symptoms peaks around the age of 15, with over 10 % of the later diagnosed cases showing first symptoms by the age of 14 (Solmi et al., 2022). The hypothesised link between MDD and AD further raises the possibility that, in childhood and adolescence, overlapping neurodevelopmental mechanisms may link genetic risk for both disorders to a similar profile of psychological dysfunction, likely stemming from heightened susceptibility to stress (for a related argument, see Dafsari and Jessen, 2020). Such a line of reasoning dovetails nicely with recent proposals that a shared neuropathological substrate of both AD and MDD is disrupted synaptic transmission in prefrontal cortex (PFC) systems (Yan and Rein, 2022), which are known to contribute substantially to successful coping across development (Vink et al., 2020). During adolescence, the PFC undergoes substantial maturational changes which are generally related to improvements in cognitive control-relevant functions, including responses to stress (Constantinidis and Luna, 2019, Vink et al., 2020). Nonetheless, alterations in the developmental pace of PFC systems can derail the typical upward trajectory of cognitive control processes. Specifically, accelerated maturation can hinder protracted fine-tuning, which often extends into the third decade of life (Sydnor et al., 2021, Tooley et al., 2021). Complementarily, maturational delays can accentuate the functional imbalance between reward sensitivity and cognitive control processes, thereby heightening risk for global psychopathology through impaired self-regulation (McTeague et al., 2017, Vink et al., 2020). Taken together, the above findings imply that understanding how MDD/AD genetic risk and exposure to early life adversity influence the developmental pace of PFC circuits could prove critical not only for intervening in adolescence/young adulthood, but also for designing longer-term programmes to delay or even prevent dementia onset in older adulthood.

To characterise the joint impact of early life adversity exposure and genetic vulnerability on psychological functioning, a network neuroscience approach with well-demonstrated utility in modelling trajectories of both normative brain maturation and (transdiagnostic) pathology was applied to longitudinal multimodal data from the Adolescent Brain and Cognitive Development (ABCD) study (Casey et al., 2018; de Lange et al., 2019; Ely et al., 2021; Fornito et al., 2015; Jones et al., 2016; Tooley et al., 2022; Váša et al., 2020; Wu et al., 2021). The objective was to elucidate the mesoscale (functional connectomic) and molecular (neurochemical and transcriptomic) neurodevelopmental patterns likely to mediate the joint effect of early life adversity and genetic vulnerability on adolescent adjustment (see Fig. 1 for a schematic representation of our model). The two types of maturational indices provide unique insights. Specifically, functional connectomic patterns, particularly those based on task-related data, are most closely linked to cognition and behaviour (cf. Dhamala et al., 2022), thereby enabling us to extend prior multiscale characterisations of structural brain development (e.g., Akarca et al., 2021). As for molecular indices of brain maturation, neurochemical correlates offer an indirect test of the proposal that disrupted synaptic transmission in the PFC is a shared neuropathological feature of both MDD and AD (Yan and Rein, 2022). Specifically, we were able to investigate the relevance of the implicated neurotransmitter systems (cf. Yan and Rein, 2022) to the functional connectomic patterns related to genetic risk for MDD/AD. Complementarily, transcriptomic correlates allowed us to probe the hypothesis that greater stress susceptibility is a neurobiological mechanism linking MDD in adolescence and young adulthood to AD risk in older age (De Jager et al., 2021, Harerimana et al., 2022).

**Fig. 1:**
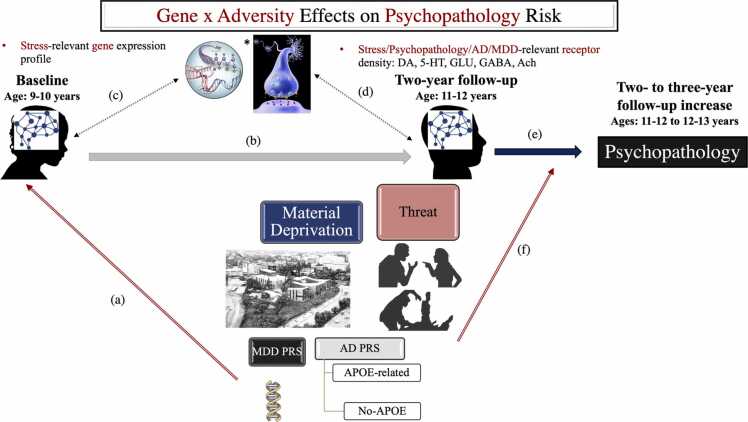
Schematic representation of our conceptual model. Genetic risk for AD (APOE vs no-APOE-based, see Method for details) and MDD, respectively, were predicted to interact with exposure to different dimensions of adversity (deprivation vs threat) to impact functional brain maturation, specifically, functional network segregation and cross-context differentiation, as observed on inhibitory control and incentive processing tasks. The objective was to identify neurodevelopmental patterns (paths a, b) and their molecular (transcriptomic and chemoarchitectural) correlates (paths c, d) that could explain the joint impact of adversity and genetic vulnerability on rising psychopathology risk in adolescence (path e). The potential for adversity/genetic risk to moderate the link between neurodevelopment and psychopathology (path f) was also probed. DA= dopamine. 5-HT = serotonin. GLU= glutamate. GABA = gamma-aminobutyric acid. Ach = acetylcholine.

Functional connectomic patterns were estimated in reference to two mental processes, inhibitory control and incentive processing, which change substantially in adolescence (Cai et al., 2021; Constantinidis and Luna, 2019; Diamond, 2013; Eckstein et al., 2022). The two are highly susceptible to adversity and predictive of MDD risk from childhood to later adulthood (i.e., >55 years), with midlife (∼55 years of age) interactions between reward and cognitive control processes further foreshadowing dementia vulnerability in older age (Cao et al., 2021, Costi et al., 2021, Ely et al., 2021, Grahek et al., 2019, Jenkins et al., 2021, Lloyd et al., 2022, McTeague et al., 2017, Ng et al., 2019; Romer and Pizzagalli, 2021; Tozzi et al., 2020; Xiao et al., 2022).

Although strongly inter-related, cross-species evidence suggests that inhibitory control and incentive processing are supported by distinguishable neural circuits (Fustiñana et al., 2021, Nougaret et al., 2022). This circuit-level specialisation emerges with maturation and affords significant benefits for cognitive performance in adulthood (Petrican and Grady, 2017, Reineberg et al., 2022, Tang et al., 2019). We therefore used the degree of cross-task reorganisation in connectivity patterns as a marker of brain maturation.

A second measure quantified levels of functional network segregation or differentiation according to the degree of within-network, relative to between-network, connectivity during each task context. This is a well-documented indicator of processing efficiency, which fluctuates with age (from birth to older adulthood) and exposure to adversity in childhood and adolescence, while also showing some sensitivity to MDD/AD clinical status (Ewers et al., 2021, Grayson and Fair, 2018, Javaheripour et al., 2021, Liu et al., 2021; Sporns and Betzel, 2016; Tooley et al., 2021). Our two network measures thus provided complementary information. Specifically, cross-task functional network reorganisation sheds light on how the brain adaptively reconfigures during different task contexts, whereas within-task segregation captures the degree of functional specialisation during performance of specific tasks. We used whole-brain analyses to comprehensively evaluate how different networks behaved across the distinct task contexts. This decision was motivated by the widespread neuropathological signature of both AD and MDD (Ewers et al., 2021, Javaheripour et al., 2021, Liu et al., 2021), as well as the extensive reorganisation and the relative “fuzziness” of functional network boundaries during childhood and adolescence (Grayson and Fair, 2018, Tooley et al., 2022).

Chemoarchitectural correlates of functional connectomic maturation were characterised in a data-driven manner in reference to five neurotransmitters which had been robustly associated with incentives, cognitive control, AD, and/or MDD. Dopamine (DA) was among those scrutinised given its contribution to incentive processing, cognitive control and motivation to exert it, as well as its role in discriminating vulnerable from resilient phenotypes following adversity exposure (Fox and Lobo, 2019; Jia et al., 2021; Karalija et al., 2021; Parr et al., 2022; Sarno et al., 2022; Westbrook et al., 2020; Westbrook et al., 2021; Zayed et al., 2022). Serotonin (5-HT) was a second neurotransmitter of interest, given its pivotal involvement in the neuropathology of MDD, its relevance to MDD-associated cognitive control and flexibility deficits, as well as contribution to remission (Reed et al., 2022, Svensson et al., 2021, Toenders et al., 2022). Glutamate (GLU) and gamma-aminobutyric acid (GABA) were also examined because of their susceptibility to adversity exposure, as well as their key relevance to the neurocognitive pathology of both MDD and AD, in particular the associated cognitive control deficits (DiSabato et al., 2021, Englund et al., 2021, Fogaça et al., 2021; Hare and Duman, 2020; Ho et al., 2021; Joffe et al., 2022; Kim et al., 2022; Prévot and Sibille, 2021; Tang et al., 2021; Tochitani et al., 2021; Yan and Rein, 2022). Finally, we probed the neurodevelopmental relevance of cholinergic receptor density maps in light of compelling evidence implicating acetylcholine (ACh) in cognitive control processes, broadly, and in their dysregulation during early AD stages, more specifically (Ballinger et al., 2016, Zhong et al., 2022).

Extant developmental theory and evidence suggest that immediately adaptive responses to adversity, such as accelerated maturation of cognitive control systems involved in emotion regulation (Brieant et al., 2021, Callaghan and Tottenham, 2016, Gee et al., 2013), can incur long-term costs, most likely because they tend to be associated with increased allostatic load and hinder the fine tuning of the slower developing association systems transdiagnostically involved in psychopathology (Colich et al., 2020, McLaughlin et al., 2020, McTeague et al., 2017, Tooley et al., 2021). It seems though plausible that the severity of environmental and genetic risk factors would moderate the psychological impact of their associated neurodevelopmental alterations. Specifically, among individuals with greater environmental/genetic vulnerability, alterations in normative patterns of brain maturation may be beneficial, at least in the short-term, as they may facilitate adjustment to inauspicious conditions, a prediction which we set out to probe in our study (cf. Fig. 1-f).

## 1 Methods and measures

**Fig. 2:**
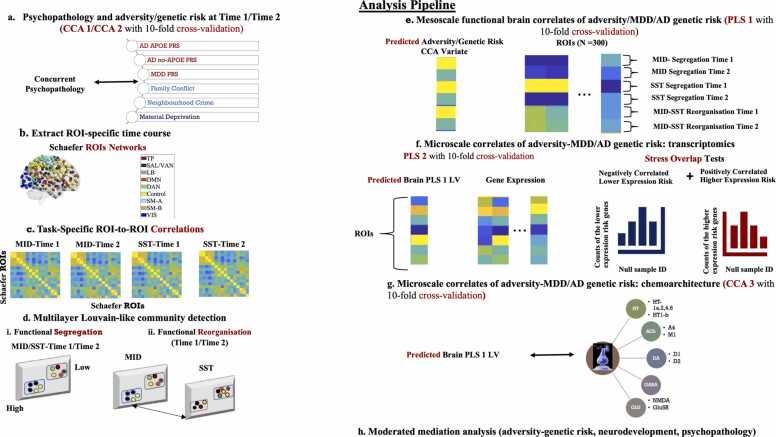
Schematic representation of our analysis pipeline. Panel (a): Two sets of CCAs with 10-fold cross-validation were conducted in order to characterise the relevance of the adversity and MDD/AD-related genetic risk factors for psychopathology at Time 1 and Time 2, respectively. Panel (b): Activation time courses were extracted for each of the 300 ROIs in the Schaefer atlas on each of the two tasks at Time 1 and Time 2, respectively. Panel (c): ROI-to-ROI correlations in time series in each task for each time point were expressed as Fisher’s transformed z-scores. Panel (d): Graph theoretical tools, including a Louvain-like multi-layer community detection algorithm, were used to estimate ROI-specific patterns of functional network segregation and task-related reorganisation. Panel (e): A PLS analysis with 10-fold cross-validation quantified the association between the predicted value of the adversity-MDD/AD genetic risk variate and ROI-specific patterns of functional network segregation and task-related reorganisation. Panel (f): A PLS analysis with 10-fold cross-validation was conducted on the ROI-specific gene expression data and the predicted PLS 1 brain LV value in order to identify its transcriptional signature. Subsequently, permutation-based testing (see main text) was used to characterise the overlap between the gene expression profile relevant to the PLS 1 brain LV and the stress susceptibility genes identified in prior studies. Panel (g): A CCA with 10-fold cross validation quantified the association between the predicted value of the PLS 1 brain LV and receptor density maps relevant to incentive processing, inhibitory control, psychopathology, as well as MDD/AD. Panel (h): A moderated mediation analysis tested whether the role of the PLS 1 brain LV in mediating the link between adversity/genetic risk and rising psychopathology varies beween the higher vs lower vulnerability individuals (cf. Fig. 1-f).

Fig. 2 contains a schematic representation of our analysis pipeline which we detail below.

### 1.1 Participants

The sample included biological parents and offspring who participated in the ongoing Adolescent Brain Cognitive Development (ABCD) study (for a detailed sample description, see Garavan et al., 2018). The analyses were based on data from Caucasian participants only because most polygenic risk markers to date have been based on this population and there is some evidence that genetic architecture and risk loci may show some racial differences (Abdellaoui and Verweij, 2021, Nievergelt et al., 2019, Wojcik et al., 2019).

The present research uses baseline, two-, and three-year follow-up data downloaded in November 2021 as part of the ABCD Study Curated Annual Release 4.0 (https://data-archive.nimh.nih.gov/abcd). Our analyses only included biologically unrelated participants whose functional neuroimaging data relevant to inhibitory control and incentive processing were recommended for inclusion by the ABCD study team, as recorded in relevant files from the 4.0 Annual Data Release. Specifically, global information about the data recommended for inclusion by the ABCD team was recorded as a binary decision (0 [“No”]/1 [“Yes”]) for each scan type (structural vs resting state vs task-related) and task at each available wave in the “abcd_imgincl01” file. Task run-specific information was recorded as “fail/pass/unspecified/no value” under ”qc_outcome” in the file “fmriresults01”. We only used data from participants who had passed the qc for all 8 relevant task runs (2 runs/task [MID/SST] at baseline and two-year follow-up respectively. Table S1 contains demographic and vulnerability-relevant information on participants included in our analyses and those excluded because of ethnicity, data quality and biological relatedness.

Our final sample included 980 Caucasian participants (470 female), the majority of whom were predominantly right-handed (N = 814). At baseline, participants were aged 9–10 years (M = 119.97 months, SD = 7.40 months). Follow-up sessions were similarly spaced across participants (baseline to two-year follow-up: M = 23.93 months, SD = 1.49 months [age range: 11–12 years, M = 143.9 months, SD = 7.65 months]; two- to three-year follow-up: M = 11.20 months, SD = 1.95 months [age range: 12–13 years, M = 155.09 months, SD = 7.58 months]).

### 1.2 Global psychopathology risk

Global psychopathology risk was quantified with the Total Problems score derived from the parent-report version of the Child Behaviour Checklist **(**CBCL, Achenbach, 2009), which is available in the ABCD 4.0 Data Release. This 112-item instrument uses a 3-point Likert rating scale (0 = not true, 1 = somewhat or sometimes true, 2 = very true or often true) to gauge participants’ psychological functioning in the prior six months. This measure was completed annually by the participating parent. For our analyses, we only used the Total Problems scores derived from the two- and three-year follow-up assessments. The Total Problems score was computed as a sum of the response values on all items (no missing values were recorded in our sample). In line with extant guidelines (Achenbach, 1991, 2013), raw scores (rather than t-scores) were used given their greater precision particularly at the extremes of the scale. In the present study, this strategy was deemed particularly appropriate, since chronological age and biological sex were controlled for in all analyses. The Total Problem scale showed excellent reliability both at the two- and three-year follow-up (Cronbach’s alphas of.94 at both timepoints).

### 1.3 Genetic risk for MDD and AD

Genetic vulnerability was quantified with polygenic risk scores (PRS, Young et al., 2019) derived from genome-wide association studies (GWAS). MDD and AD PRSs were each computed as the weighted sum of risk alleles based on the summary statistics of two large GWASs focused on each disorder (Howard et al., 2019; Kunkle et al., 2019, for MDD and AD, respectively), which had been made available by the original authors via the “Public Results” tab on the FUMA website (https://fuma.ctglab.nl/browse, Watanabe et al., 2017). We focused on these two GWASs for the following reasons: (1) match to our Caucasian-only sample with Howard et al., in particular, being the largest MDD GWAS to date of this population; (2) use of clinical diagnosis-based patient cases and, for Kunkle et al., confirmed clinical cases of late-onset sporadic AD, which would ease the interpretation of the derived PRSs and associated findings.

For AD, we computed a separate APOE region (chromosome 19:44.4–46.5 Mb) only- vs no-APOE region PRS (cf. Leonenko et al., 2019) because the two PRSs forecast distinguishable trajectories of neurocognitive impairments and differential susceptibility to environmental factors (no-APOE-based > APOE-based PRS) to environmental factors (Frisoni et al., 2022, Martens et al., 2022). APOE-based risk is associated with neurodevelopmental deviations from infancy onwards (Dean et al., 2014) and is mainly predictive of memory deficits, which stem from (medial) temporal and posterior parietal atrophy (Frisoni et al., 2022). Complementarily, no-APOE based AD PRS forecasts a profile of relatively greater deficits in cognitive control, language and visuospatial processing, reflecting a much more extensive neurodegenerative pattern encompassing temporal, frontal and parietal lobe structure (Frisoni et al., 2022).

For MDD, we used the top 10k most informative variants, based on approximately 76k patients and 230k, which had been made publicly available by Howard et al. on FUMA. These variants had been obtained by clumping the corresponding GWAS statistics with the following parameters p1 = p2 = 1, window size < 500 kb, and r^2^ > 0.1.

To compute MDD and AD PRSs based on the * .genotype ABCD data (collected at baseline), we used the PLINK genetic analysis toolset (Chang et al., 2015) with SNPs significant at GWAS level *p* ≤ 5 × 10^-8^. We believe that our SNP filtering criteria were well-justified since GWAS significant SNPs are likely to make the most robust contribution to disease risk and, even for AD, 24 of the 28 risk loci that survived preprocessing (see below) had an associated GWAS level p < 5 × 10^-8^. However, in supplemental analyses, we confirmed that all the environmental/genetic risk patterns herein identified also emerge when using more lenient *p*-thresholds (see Figs. S1–2).

Prior to PRS computation, the following preprocessing steps were implemented: (1) genes with a minor allele frequency (MAF) < 0.05, insertion/deletion and ambiguous single nucleotide polymorphisms (SNPs) (i.e., A/T and G/C pairs) were excluded; (2) highly correlated SNPs (r^2^ >0.10) within a 500 kb window were eliminated. The SNPs which survived the preprocessing and had an associated GWAS level *p* ≤ 5 × 10^-8^ contributed to the computation of the disorder-specific PRSs (MDD PRS: N = 8 SNPs; no-APOE AD PRS: N = 10 SNPs; APOE AD PRS = 14 SNPs).

### 1.4 Postnatal adversity

Although the adversity scales described below were completed on an annual basis, we only analysed the baseline and two-year follow-up responses because of their contemporaneity with the neuroimaging assessments. Material deprivation was evaluated only by the parent, while psychosocial adversity was estimated by both youth and their caregiver. However, because we sought to gauge participants’ conscious awareness of domestic violence and neighbourhood crime, our analyses included only the youth’s responses on the corresponding scales.

#### 1.4.1 Material deprivation

Financial deprivation was assessed with a 7-item scale developed by Diemer et al. (2012) to assess unmet material needs in the areas of housing, food and medical care in the 12 months preceding assessment. Each item is scored as 1 or 0 (yes/no). Their responses to all items were averaged separately for baseline/Time 1 (M = 0.26 [SD = 0.80]; Cronbach’s alpha of.72) and the two-year follow-up/Time 2 (M = 0.23 [SD = 0.75]; Cronbach’s alpha of.71). Higher scores indicated experiences of greater financial hardship.

#### 1.4.2 Family conflict

The 9-item Family Conflict subscale of the Moos Family Environment Scale (Moos and Moos, 1994) gauged exposure to domestic violence. Each item is scored as 1 or 0 for true/false, with reverse coding of items that imply lack of conflict in the home (e.g., “We fight a lot in our family.” versus “Family members rarely become openly angry.”). Higher scores indicate a more conflictual family environment. The youth version of the scale demonstrated acceptable reliability at both waves of interest (Cronbach’s alphas of.65 [M = 0.21, SD = 0.20] and.66 [M = 0.18, SD = 0.20] for baseline and two-year follow-up, respectively).

#### 1.4.3 Perceived neighbourhood crime and safety

The 3-item Neighbourhood Safety/Crime Scale uses a five-point Likert Scale (5 = strongly agree to 1 = strongly disagree) to gauge perceptions of threat related to the neighbourhood in which the respondent resides (i.e., areas within a 20-minute walk from the respondent’s home, Echeverria et al., 2004). At both time points, the youth completed only a 1-item version of the scale (“My neighbourhood is safe from crime”; M = 4.22 [SD = 0.93] and M = 4.22 [SD = 0.90] for baseline and two-year follow-up, respectively). Higher scores on this scale indicate greater neighbourhood safety. Prior to analysis, scores were reverse coded in order to create an index of neighbourhood crime.

### 1.5 Functional neurodevelopment

The functional neuroimaging assessments described below were collected at baseline and the two-year follow-up.

#### 1.5.1 fMRI tasks

In both tasks described below, task parameters are dynamically adjusted to maintain a set accuracy level across participants (Casey et al., 2018). Thus, given their limited informational value, behavioural performance indices were not entered in any of our analyses.

**Inhibitory control.** An in-scanner Stop Signal Task (SST) measured the ability to inhibit an ongoing speeded motor response to a “Go” signal (Logan, 1994). In ABCD, the task comprises two runs of 180 trials each: 150 “Go” trials, 15 “Stop” trials expected to be unsuccessful and 15 “Stop” trials expected to be successful. To maintain the breakdown of the successful/unsuccessful “Stop” trials, a tracking algorithm was implemented to alter the interval between the presentation of the ‘Go stimulus’ and the onset of the ‘Stop’ signal based on the participant’s performance. Each run was restricted to begin with a ‘Go’ trial and stop trials were separated by a minimum of one ‘Go’ trial (for further details on this task, including criticisms pertaining mostly to behavioural data analyses, see Bissett et al., 2021; Casey et al., 2018; Garavan et al., 2018).

**Incentive processing.** A monetary incentive delay task gauged anticipatory and consummatory reactions to rewards and losses, as well as drive to engage in speeded responses for monetary gains or avoidance of losses (Casey et al., 2018; Knutson et al., 2000). Each of the two task runs contains 50 contiguous trials consisting of a monetary incentive cue presented for 2000 ms (10 of each type: Win $.20, Win $5, Lose $.20, Lose $5, $0-no money), a variable (1500–4000 ms) anticipation period, a response-to-target interval (150–500 ms), followed by feedback on the outcome of the trial (2000 ms – target duration). In total, there are 40 reward, 40 loss and 20 no-money trials.

#### 1.5.2 MRI data acquisition

Scanning was performed across 21 US sites, with a protocol harmonised for Siemens Prisma, Philips, and GE 3T scanners (for details, see Casey et al., 2018; Hagler et al., 2019). Scanner type was controlled for in all analyses by using site id as a covariate to account for magnet and sociodemographic differences among sites (Chaarani et al., 2021; Rosenberg et al., 2020). T1-weighted were acquired with an MPRAGE-PMC (Prospective Motion Correction) sequence (TR=2500 (Siemens/GE)/6.31 (Phillips) ms, TE = 2.88 (Siemens)/2.9 (Phillips)/2 (GE) ms, flip angle = 8°, FOV = 256 × 256 mm (Siemens/GE)/256 × 240 (Phillips), 176 (Siemens)/225 (Phillips)/208 (GE) slices of 1 × 1 mm in-plane resolution, 1 mm thick). The fMRI data were acquired with a multiband EPI sequence (TR = 800 ms, TE=30 ms, flip angle = 52°, FOV = 216 ×216 mm, 60 slices of 2.4 × 2.4 mm in-plane resolution, 2.4 mm thick, multiband acceleration factor of 6).

#### 1.5.3 fMRI data preprocessing

Our analyses used minimally preprocessed fMRI data available as part of the ABCD Study Curated Annual Release 4.0. The minimal preprocessing pipeline involved correction for head motion (Cox, 1996), spatial and gradient distortions (Andersson et al., 2003; Jovicich et al., 2006), bias field removal, and co-registration of the functional images to the participant’s T1– weighted structural image. We further applied the following steps: (1) elimination of initial volumes (8 volumes [Siemens, Philips], 5 volumes [GE DV25], 16 volumes [GE DV26] to allow the MR signal to reach steady state equilibrium, (2) linear regression-based removal of quadratic trends and 24 motion terms (i.e., the six motion parameters, their first derivatives, and squares, cf. Power et al., 2015) from each voxel’s time course.

#### 1.5.4 ROI definition

Our main analyses were based on the Schaefer 300 parcel-functional atlas (Schaefer et al., 2018, Yeo et al., 2011), downloaded from https://github.com/ThomasYeoLab/CBIG. The atlas version we used encompasses 17 functional networks, spanning core systems, such as the DMN (A/B/C), Control (A/B/C), Salience/Ventral Attention (A/B), Dorsal Attention (DAN A/B), Somatomotor (SM A/B), Visual (VIS Central/Peripheral), Limbic (LB A/B), and Temporo-parietal (TP). The Schaefer atlas was available in the Montreal Neurological Institute (MNI) standard space. To align it with the participants’ native space for each of the four tasks (SST at Time 1/Time 2, MID at Time 1/Time 2), the following steps were implemented in FSL: (1) the middle image in each task run was converted to the MNI space (via the MNI-152 brain template available in FSL) and the inverse transformation (MNI-to-participant native space) was estimated; (2) the inverse transformation was used to align the Schaefer atlas to each participant’s functional images, separately for each task run (SST at Time 1/Time 2, MID at Time 1/Time 2).

#### 1.5.5 ROI-to-ROI correlations in timeseries

Pairwise Pearson’s correlations between all the Schaefer ROIs were computed separately for the SST and MID in Matlab and expressed as Fisher’s z-transformed scores. Because the two tasks are very similar in duration, we used all available data from each. Similar to prior studies using multilayer community detection algorithms (e.g., Finc et al., 2020), only the positive Fisher’s z-scores were entered in the network-level analyses detailed below, while negative z-scores were set to zero.

#### 1.5.6 Network-level analyses

All the network-level metrics were computed using the Brain Connectivity Toolbox (BCT, Rubinov and Sporns, 2010) and the Network Community Toolbox (NCT, Bassett, D.S. [2017, November]. Network Community Toolbox. Retrieved from http://commdetect.weebly.com/), as described below.

To characterise patterns of ROI-based functional network segregation and reorganisation between SST and MID, we used a multilayer generalised Louvain-like community detection algorithm, first introduced by Mucha et al. (2010) and implemented in the NCT. This algorithm partitions a network with multiple layers into non-overlapping groups of nodes (i.e., functional communities) with the goal of maximising an objective modularity quality function, defined as

$$Q=\frac{1}{2\mu }\sum_{ijlr}\left[(w_{ijl}-\gamma_{l}e_{ijl})\delta_{ir}+\delta_{ij}\omega_{jlr}]\delta (g_{il},g_{jr}\right)$$

where 2μ is the sum of all connection weights in the network across all layers, w_ijl_ represents the connection strength between nodes i and j in layer l; γ_l_ is a resolution parameter determining the size of the identified modules in layer l; e_ijl_ is the connection strength expected by chance between nodes i and j in layer l, and defined as $e_{ijl}=\frac{s_{il}s_{jl}}{v}$ with s_il_ and s_jl_ being the sum of all connection weights of node i and j, respectively, in layer l, while v is the sum of all connection weights in the network in layer l; ω_jlr_ is the connection strength between node j in layer l and node j in layer r, and g_il_ and g_jr_ give the community assignments of node i in layer l and node j in layer r.

In the above modularity quality optimisation, there are two free parameters, the spatial resolution parameter, γ, which tunes community size within each layer, and the cross-layer connection strength parameter, ω, which determines community stability across layers. In line with extant practices (Finc et al., 2020; Mattar et al., 2015), the spatial resolution parameter was set to the default value of 1. Taking our cue from other investigations of heterogenous mental states (Finc et al., 2020), we set to 0.5 the cross-layer (MID-SST at Time 1; MID-SST at Time 2; Time 1-to-Time 2 MID; Time 1-to-Time 2 SST) connection strength parameter. To account for the near degeneracy of the modularity landscape (Good et al., 2010), the multilayer community detection algorithm was initiated 100 times and all the functional network interactions indices detailed below were averaged across all iterations.

**Functional network development.** Patterns of functional network interactions were estimated through two ROI-level functional interaction diagnostics, which were computed in the NCT across the 100 iterations of the multilayer community detection algorithm: (1) functional flexibility/cross-task functional reorganisation, operationalised as the number of times each ROI in the Schaefer atlas changed communities between MID and SST at each wave, and (2) differentiation/segregation (called “recruitment” in the NCT), which was estimated separately for MID vs SST at each wave, and operationalised as the number of times a given ROI was assigned to the same community as the other ROIs in its native functional system, as defined in the Schaefer functional atlas.

### 1.6 Gene expression profiles relevant to functional neurodevelopment

To identify gene expression profiles relevant to functional neurodevelopment, we used micro-array gene expression data from six postmortem brains (1 female, ages 24.0–57.0, 42.50 +/- 13.38) provided by the Allen Institute for Brain Science (https://www.brain-map.org/). All six brains had left hemisphere data, but only two brains contained data from the right hemisphere. Based on observations that gene expression patterns are largely symmetric across the two hemispheres, our main analyses focused on gene expression patterns mirrored across the two hemispheres. This strategy ensured comparability with the remaining whole-brain analyses (for a similar strategy, see Petrican et al., 2022). However, in supplemental analyses, we verified that all the results are replicated when using only the left-hemisphere ROIs (cf. Ball et al., 2020; Hansen et al., 2021; see Fig. S5-a).

The gene expression data was processed with abagen (Markello et al., 2021). Microarray probes were reannotated based on the probe-to-gene mapping information provided by Arnatkevičiūtė et al. (2019) and filtered based on their expression intensity relative to background noise (Quackenbush, 2002), such that probes with intensity less than the background in > = 50 % of samples across donors were discarded. When multiple probes indexed the expression of the same gene, we selected and used the probe with the most consistent pattern of regional variation across donors (i.e., differential stability; Hawrylycz et al., 2015). The MNI coordinates of tissue samples were updated to those generated via non-linear registration using the Advanced Normalisation Tools (ANTs; https://github.com/chrisfilo/alleninf). Samples were assigned to brain regions in the Schaefer atlas if their MNI coordinates were within 2 mm of a given parcel. All tissue samples not assigned to a brain region in the provided atlas were discarded.

Inter-subject variation was addressed by normalising tissue sample expression values across genes using a robust sigmoid function (Fulcher et al., 2013):

xnorm=1/(1+exp(-(x-〈x〉)/IQRx))

where 〈x〉 is the median and IQR*x* is the normalised interquartile range of the expression of a single tissue sample across genes. Normalised expression values were then rescaled to the unit interval:

xscaled=(xnorm−min(xnorm))/(max(xnorm)−min(xnorm))

Gene expression values were then normalised across tissue samples using an identical procedure. Samples assigned to the same brain region were averaged separately for each donor and then across donors. After we eliminated the ROIs without reliable gene expression (based on the abagen parameters outlined above), the resulting gene expression matrix, used in all our analyses, was in the format 297 (ROIs) x 15,632 (genes). In this matrix, each cell contained the normalised expression level of a gene as observed in one of the ROIs from the Schaefer atlas. A list of ROIs lacking reliable gene expression is included in the Supplemental Materials (Table S2).

### 1.7 Receptor density maps relevant to functional neurodevelopment

To estimate receptor density maps for our neurotransmitters of interest (Ach, DA, GABA, GLU, HT), we used the normative atlas put together by Hansen et al. (2022), which is based on positron emission tomography (PET) data from over 1200 healthy individuals. Group-averaged pre-processed PET images corresponding to each tracer map for Ach, DA, GABA, GLU, HT were downloaded from https://github.com/netneurolab/hansen_receptors/tree/main/data/PET_nifti_images. Each tracer map was parcellated in the MNI-152 space based on the Schaefer atlas using the “Parcellater” function from neuromaps (Markello et al., 2022). Following Hansen et al., 2021, Harerimana et al., 2022 strategy, an adapted version of the “make_receptor_matrix.py” script (https://github.com/netneurolab/hansen_receptors/tree/main/code) was employed to estimate the weighted average of the receptor density maps corresponding to each neurotransmitter of interest. This was done separately for each tracer based on the number of participants in the respective study (see Table 1 in Hansen et al., 2022).

**Table 1:** Demographic, adversity and genetic risk information for the entire sample, as well as the low and high vulnerability participants.

Variable	Total N = 980	Low vulnerability N = 490	High vulnerability N = 490	Mann-Whitney U test standardised test statistic (*p*-value)
Age (years)*	10.99 ± 0.62	11.01 ± 0.64	10.98 ± 0.61	0.740 (0.459)
Sex (F/M)	470/510	243/247	227/263	1.023 (0.307)
Handedness (% mostly right-handed)	83 %	83 %	83 %	0.170 (0.865)
Material Deprivation^a^	.24 ± 0.68	0.03 ± 0.16	0.46 ± 0.90	11.388 (0.001)
Family Conflict^a^	.20 ± 0.17	0.12 ± 0.11	0.27 ± 0.18	14.891 (0.001)
Neighbourhood Safety^a^	4.22 ± 0.76	4.59 ± 0.51	3.85 ± 0.78	15.785 (0.001)
Psychopathology Risk^b^	14.70 ± 13.52	12.48 ± 12.43	16.92 ± 14.20	5.662 (0.001)
APOE AD PRS	-0.011 ± 0.031	-0.007 ± 0.031	-0.015 ± 0.029	3.632 (0.001)
No-APOE PRS	0.011 ± 0.012	0.007 ± 0.011	0.016 ± 0.012	11.398 (0.001)
MDD PRS	0.004 ± 0.002	0.003 ± 0.002	0.004 ± 0.002	6.109 (0.001)

Note. Because of their robust correlations (*r*s from.23 to.77) across the full sample and the two subsamples, measures were averaged across all the analysed waves, ^a^ baseline to two-year follow-up, ^b^ two- to three-year follow-up. The Mann-Whitney U test was used to compare the high and low vulnerability groups on the variables included in Table 1.

Each receptor density measure was standardised across all the parcels in the Schaefer atlas. Four of the five indices of HT receptor density (HT1a, HT2, HT4, HT6) were moderately to strongly correlated (*r*s from.28 to.57). Consequently, we averaged their standardised scores, while the density index corresponding to HT1b was entered separately in the analyses.

### 1.8 Analytical tools

#### 1.8.1 Canonical correlation analysis (CCA)

To probe the relationship between psychopathology and adversity/genetic risk factors, as well as between patterns of functional network development and receptor density maps, we conducted three canonical correlation analyses (CCAs) with cross-validation (cf. Hair et al., 2014). CCA is a multivariate technique which seeks maximal correlations between two sets of variables by creating linear combinations (i.e., canonical variates) from the variables within each set. CCA was implemented in Matlab using the canoncorr module. In line with existing guidelines (Hair et al., 1998), all CCAs were conducted on sample sizes more than 10 times greater the number of contributing variables. A 10-fold cross-validation procedure tested the performance of our CCA-derived models. For all sets of CCAs, discovery analyses were conducted on nine folds of data and the resulting CCA weights were employed to derive predicted values of the adversity/genetic (/brain network) and psychopathology (/receptor density) variate in the left-out (“test ”) fold. This procedure was repeated until each of the ten folds served as “test data” once. The correlation between the predicted variates across all testing folds was evaluated using a permutation test with 100,000 samples (cf. Smith et al., 2015).

Relationships between measured variables and their corresponding CCA-derived variate are described via correlations between the observed value of a given variable and the predicted value of its corresponding variate, as well as standardised coefficients, analogous to multiple regression coefficients, which indicate the unique association between the observed value of a variable and the predicted value of its corresponding variate. 99.9 % confidence intervals (CI) for each correlation and standardised regression-like coefficient were obtained by using the bootci function in Matlab (with default settings and 100,000 boot- strap samples). The CIs were selected to match those used in the partial least squares analyses for which clearer guidelines exist (see the following section). The aforementioned correlation and standardised regression coefficients-like are a more conservative estimate of the tradional canonical loadings and canonical weights (Hair et al., 2014), respectively, as they are estimated in the test, rather than discovery, folds.

#### 1.8.2 Partial least squares (PLS) analysis

Partial least squares correlation (i.e., PLS, Krishnan et al., 2011), a multivariate data-driven manner technique which can identify relationships between neural/gene expression patterns (latent variables or LVs) and individual differences variables (behavioural PLS), was used to probe the transcriptomic and functional brain profiles linked to AD/MDD-related genetic vulnerability and adversity exposure. PLS was implemented using a series of Matlab scripts, which are available for download at https://www.rotman-baycrest.on.ca/index.php?section= 345.

Two behavioural PLS analyses were conducted. In the first analysis, the “behavioural” set included the adversity/genetic vulnerability CCA variate linked to psychopathology. The brain matrix contained the ROI-specific indices of functional reorganisation and segregation estimated in NCT and modelled as six separate conditions: SST-MID reorganisation at Time 1/Time 2, MID segregation at Time 1/Time 2, and SST segregation at Time 1/Time 2.

The second PLS analysis sought to characterise the transcriptomic signature of the functional neurodevelopmental patterns linked to adversity/genetic vulnerability in the first PLS analysis. Specifically, it estimated the correlation between the brain LV identified in behavioural PLS analysis 1 and the ROI x gene expression level matrix derived with the abagen toolbox from the transcriptomic maps provided by the Allen Brain Institute.

In both PLS analyses, potential axis rotations (i.e., changes in the order of the extracted LVs) and reflections (i.e., changes in the sign of the saliences), which may occur during resampling with either permutations, were corrected with a Procrustes rotation, which defines a transformation through which the resampled singular value decomposition outcome (i.e., the identified LVs) is rotated to match most closely the original singular value decomposition outcome (Milan and Whittaker, 1995).

**Generalisability and significance testing**. The generalisability of our PLS models was tested using a 10-fold cross-validation procedure, similar to the one implemented for the CCAs. Specifically, the PLS analysis was run on nine folds of data and the weights associated with identified brain (PLS analysis 1)/gene (PLS analysis 2) LV were used to compute the predicted value of the brain/gene LV in the test fold. This procedure was repeated until each of the ten folds served as “test data” once.

In the brain PLS analysis, the significance of the extracted LVs was estimated in the test folds by.

(1) multiplying the ROI measure in each condition (see Fig. 4) by its corresponding weight (as extracted in the training folds);

**Fig. 3:**
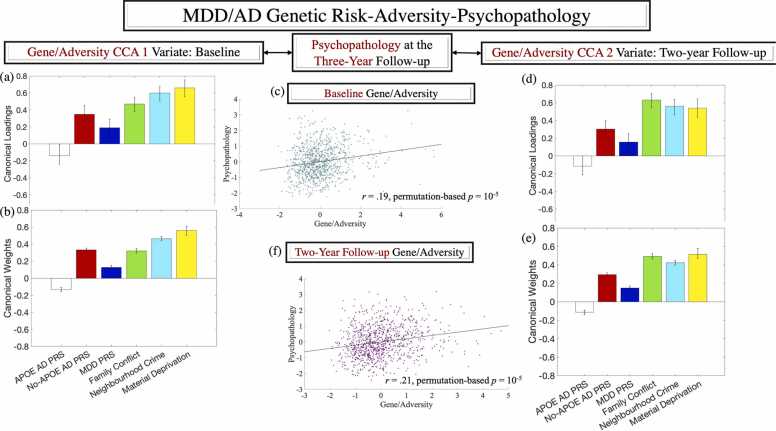
Correlation [panels a, d] and standardised coefficients [panels b, e] describing the relationship between the observed adversity/genetic risk variables and the predicted value of their corresponding canonical variate across all test CCAs. Panels (c) and (f) contain the scatter plot describing the linear relationship between predicted value of the two variates at baseline and the two-year follow-up, respectively. Error bars are based on the bootstratpping procedure (99.9% confidence intervals) as described in the text. CCA = canonical correlation analysis. AD = Alzheimer’s Disease. MDD = Major Depressive Disorder. PRS = polygenic risk score.

**Fig. 4:**
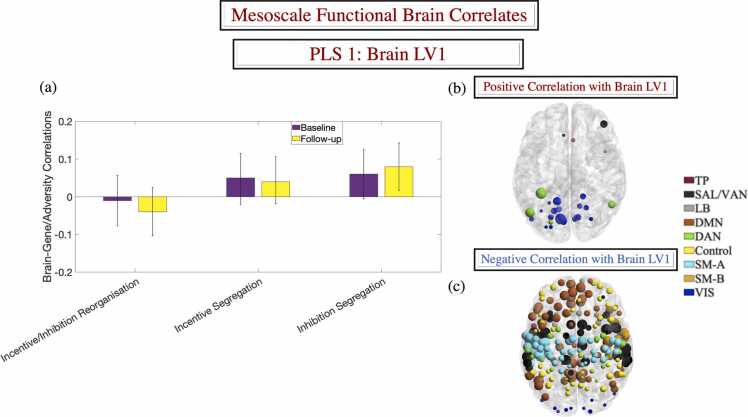
The brain LV from the behavioural-PLS analysis linking the adversity/genetic risk CCA variate to functional brain development. Panel (a) shows the correlations between the CCA variate and the predicted brain scores in each condition (based on the 10-fold cross-validation procedure). Panels (b) and (c) depict the Schaefer ROIs with robust loadings (based on cross-validated 99.9 % confidence intervals, as described in the main text) on the LV in panel (a) and visualised with the BrainNet Viewer (http://www.nitrc.org/projects/bnv/) (Xia et al., 2013). ROI colours reflect Schaefer et al.’s network assignments. In panels (b) and (c), the size of the ROIs is proportional to the strength of their correlation with the brain LV. In panel (a), error bars are the 95 % confidence intervals from the bootstrap procedure. Confidence intervals that do not include zero reflect robust correlations between the adversity/genetic risk CCA variate and the predicted brain score in a given condition across all participants. CCA = canonical correlation analysis. LV= latent variable. Schaefer networks: TP= temporo-parietal. SAL-VAN = salience/ventral attention. LB = limbic. DMN = default mode. DAN = dorsal attention. SM-A =somatomotor-A. SM-B =somatomotor-B. VIS = visual.
(2) multiplying the gene/adversity scores by their corresponding condition-wise weights (as extracted in the training folds);
(3) applying singular value decomposition (SVD) to the condition-wise correlation between the ROI and gene-adversity matrix, computed as described at (1) and (2) (cf. Krishnan et al., 2011);
(4) generate 100,000 null distributions by within-condition permutation of the gene/adversity scores and apply SVD to each pair of brain-gene/adversity matrices;
(5) count the number of null samples in which the first extracted SVD exceeded the value of the first extracted SVD from the original data (see point [3]).

In the gene PLS analysis, the significance of the extracted LVs was estimated in the test folds by.

(1) multiplying each gene by its corresponding weight (as extracted in the training folds) and summing across all genes to quantify the predicted gene LV score;
(2) estimate the correlation between the brain LV (cf. Fig. 4) and the predicted gene LV score;
(3) use Váša et al. (2017) “rotate_parcellation” function in Matlab (https://github.com/frantisekvasa/rotate_parcellation/commit/bb8b0ef10980f162793cc180cef371e83655c505) in order to generate 100,000 spatially constrained permutations of the brain LV (cf. Fig. 4);
(4) count the number of null samples in which the correlation between the brain and gene LV exceeded the absolute correlation value observed in the original data (see point [2]).

**Reliability testing.** Using the bootci function in MATLAB (with default settings and 100,000 bootstraps), we estimated the 95 % confidence intervals (CI) for the correlations between the predicted value of the brain/gene LV and the value of the variables in the “behavioural” set corresponding to the respective PLS analysis (adversity-genetic vulnerability [PLS analysis 1] and functional neurodevelopment LV [PLS analysis 2]). To estimate the contribution of each ROI (PLS analysis 1) or gene (PLS analysis 2) to the corresponding LV, we estimated the 99.9% CI of the correlation coefficient corresponding to each ROI-level index or gene expression level and the scores of the associated LV (estimated in the test fold). The thresholds used for testing the reliability of within- versus between-set associations reflect existing guidelines for PLS analyses (McIntosh and Lobaugh, 2004).

#### 1.8.3 Stress susceptibility overlap tests

To test whether the neurodevelopmental patterns related to adversity exposure and genetic risk for AD/MDD are linked to greater expression of genes implicated in susceptibility to stress and adversity, we used the eQTL analysis data from the GWAS conducted by Nagel et al. (2020). The stress-relevant candidate risk loci had been mapped onto the corresponding genes by the original authors using the SNP2GENE tool in FUMA and made available via the Public Results tab (https://fuma.ctglab.nl/browse, Watanabe et al., 2017). Based on their eQTL analysis output, we identified 72 stress-linked genes reliably expressed in our data. Of these, the risk allele(s) reduced gene expression for 27 of them (stress_low), but increased gene expression for the remaining 45 (stress_high).

To characterise the transcriptomic signature of the adversity/genetic risk-relevant brain profile, we focused on genes that showed a robust (99.9 % CI) association with their corresponding PLS-extracted LV. As an example, for the genes positively correlated with their corresponding LV, the procedure was as follows: (1) we obtained separate counts of the number of stress_low and stress_high genes, respectively (stress_low_pos and stress_high_pos, respectively); (2) we counted the number of genes positively correlated with our gene LV (Orig_pos); (3) from each of the corresponding gene LVs in the null distribution (each of which had been aligned with the original gene LVs via a Procrustes transform), we selected a number of genes equal to Orig_pos (Null_pos); (4) we counted the number of null samples (out of the total of 100,000) in which the number of either stress_low_pos or stress_high_pos in Null_Pos exceeded the one observed in Orig_pos. The same procedure was followed for the genes negatively correlated with our gene LV. Estimation of whether the identified transcriptomic signatures implied greater stress susceptibility varied depending on whether the brain and gene were positively versus negatively correlated. For example, if the gene and brain LVs were positively correlated, stress susceptibility would be estimated as a conjunction of stress_low genes negatively, and stress_high genes positively, correlated with the extracted gene LV, as observed in the original data relative to the null distribution.

#### 1.8.4 Path model

To test the role of the identified neurodevelopmental profile in mediating the impact of adversity and MDD/AD-related genetic risk on psychopathology risk among the least versus the most vulnerable adolescents, we conducted one moderated mediation analysis using Hayes’ PROCESS 4.2 macro for SPSS (Hayes, 2018). PROCESS is an ordinary least squares (OLS) and logistic regression path analysis modelling tool, based on observable variables. The moderated mediation model was tested employing 95 % CI with 50,000 bootstrapping samples. In line with extant guidelines on balancing Type I and Type II errors in mediation analyses (Hayes and Scharkow, 2013, Tofighi and Kelley, 2020), the CIs for indirect effects was estimated using percentile bootstrap, which is the default option in PROCESS 4.2. As recommended by Hayes and Cai (2007), a heterodasticity consistent standard error and covariance matrix estimator was used. Bootstrapping-based 95% CIs for the indirect effects and for the moderated mediation index (cf. Miočević et al., 2018; Walters, 2018), as outputted by PROCESS, were used as effect size estimates.

### 1.9 Control variables

In line with prior reports based on ABCD data (Chaarani et al., 2021, Modabbernia et al., 2021a, Modabbernia et al., 2021b; Rosenberg et al., 2020), we controlled for the following variables in the moderated mediation, as well as all the CCA and PLS cross-validation analyses: (1) chronological age in order to estimate accelerated/decelerated development; (2) biological sex (coded as “1” for females, “0” for males); (3) handedness (coded as “0” for right-handedness and “1” for non-right-handedness); (4) serious medical problems, which was based on the ABCD Parent Medical History Questionnaire and computed as an average of unplanned hospital visits in the prior year for chronic health conditions, head trauma, loss of consciousness and/or convulsions/trauma; (5) scanner site (21 dummy variables to account for scanner-related differences), and (6) average modality-specific motion per participant (Power et al., 2015). Thus, all the relationships herein documented were extracted based on the non-residualised variables and remained significant after controlling for the above well-known confounders. Because the difficulty of the MID and SST trials was dynamically adjusted to maintain a set accuracy level (Casey et al., 2018), we did not control for behavioural performance on these tasks.

### 1.10 Multiple testing

We conducted seven multivariate analyses based on the Schaefer atlas (the corresponding Gordon atlas-based analyses were confirmatory, only seeking to replicate the effects observed with the Schaefer atlas). These comparisons, all of which were based on out-of-sample prediction, included.

• three CCAs (see Fig. 3-c, f, Fig. 6-f),

**Fig. 5:**
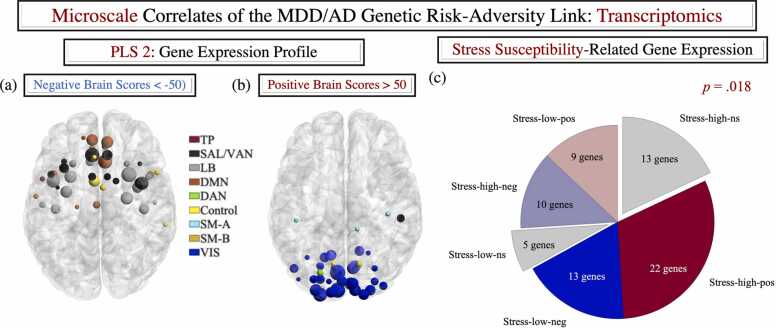
Results of the stress susceptibility overlap analyses. Panels (a) and (b) represent the spatial expression map of the gene LV identified in the gene-brain behavioural PLS analysis. For presentational purposes only, panels (a) and (b) depict only the ROIs with gene LV scores greater than 50 in absolute value (the LV scores had been centred around 0). The ROIs were visualised with the BrainNet Viewer (http://www.nitrc.org/projects/bnv/). ROI colours reflect Schaefer et al.’s network assignments and their size is proportional to how strongly they express the gene LV (i.e., the absolute value of the associated predicted gene score, as derived from the cross-validation procedure). Panel (c) depicts the results of the stress susceptibility overlap analyses. As described in the text, these are based on the gene LV depicted in panels (a) and (b). LV= latent variable. Schaefer networks: TP= temporo-parietal. SAL-VAN = salience/ventral attention. LB = limbic. DMN = default mode. DAN = dorsal attention. SM-A =somatomotor-A. SM-B =somatomotor-B. VIS = visual.

**Fig. 6:**
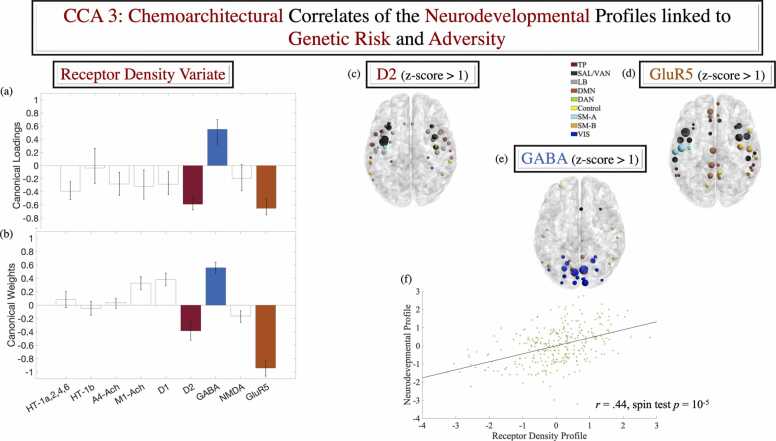
Receptor density maps linked by CCA to the adversity/genetic risk-relevant neurodevelopmental profile (cf Fig. 3, Fig. 4). Correlation [panel a] and standardised coefficients [panel b] describing the relationship between the observed receptor density maps and the predicted value of their corresponding canonical variate across all test CCAs. Panels (c)-(e) depict the D2, Glu5R and GABA receptor density maps thresholded at a z-score value > 1 for presentational purposes only. Panel (f) contains the scatter plot describing the linear relationship between predicted value of the brain LV extracted with PLS (cf. Fig. 4) and the receptor density CCA variate. The ROIs were visualised with the BrainNet Viewer (http://www.nitrc.org/projects/bnv/). ROI colours reflect Schaefer et al.’s network assignments and their size is proportional to the density of the respective receptor. Error bars are based on the bootstratpping procedure (99.9 % confidence intervals) as described in the text. CCA = canonical correlation analysis. GLU = glutamate. GABA = gamma-aminobutyric acid. D = dopamine.
• two PLSs (see Figs. 4 and 5-a, b),
• one null distribution-based analyses validating the stress susceptibility transcriptomic signature (see Fig. 5-c) and
• one moderated mediation analysis (see Fig. 7).

**Fig. 7:**
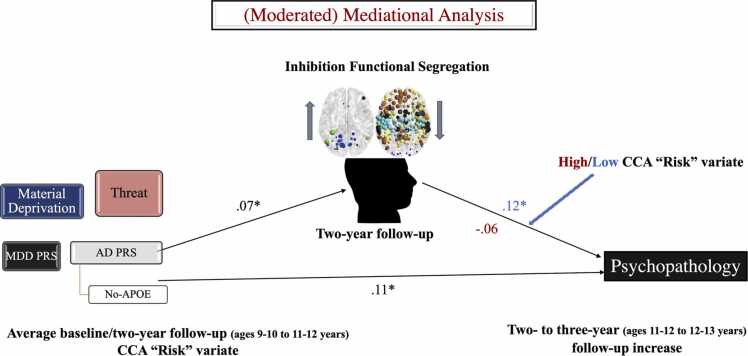
(Moderated) mediational models linking the adversity/genetic risk CCA variate to increases in psychopathology from the two- to the three-year follow-up. CCA = canonical correlation analysis. AD = Alzheimer’s Disease. MDD = Major Depressive Disorder. PRS = polygenic risk score.

There was no need to correct across regions or behavioural measures since the models were explicitly multivariate.

## 2 Results

All the results herein reported were replicated using data from the Gordon atlas (Gordon et al., 2016) (see Supplemental materials). All the reported PLS analyses had been conducted on ROI-specific indices. Network labels attached to the ROIs are included for informational purposes only.

### 2.1 Exposure to adversity and genetic risk for accelerated brain ageing are additive predictors of psychopathology risk in adolescence

Ten discovery CCAs probing the relevance of genetic risk and chronic adversity exposure to psychopathology revealed one significant mode for Time 1 and Time 2, respectively, both of which were validated across all test folds (*r*s of.19 and.21, respectively, permutation-based *p*s = 10^-5^). At both time points, the identified modes implied that chronic adversity exposure, and polygenic risk for MDD and AD (no-APOE-based), respectively, may additively raise vulnerability to psychological problems (see Fig. 3). Of note, the APOE-based AD PRS did not show a robust loading on the identified adversity-genetic risk CCA variate related to psychopathology (cf. Fig. 3-a, d).

### 2.2 Adversity exposure and genetic risk for MDD/AD are linked to altered development of the inhibitory control task architecture

A behavioural-PLS analysis identified a pattern of apparent accelerarated/delayed functional network maturation (increased/reduced functional network segregation), specific to the inhibitory control task, which covaried with the adversity-genetic risk CCA mode at Time 2 (see Fig. 4-a). This PLS profile, in which the gene/adversity scores accounted for 54 % of the variance in the brain data, was validated across all 10 test folds (*p* = .018). The extracted brain LV linked the adversity-genetic risk CCA mode to greater VIS and DAN, but weaker DMN, SM, SAL/VAN and FPC functional network segregation (see Fig. 4-b, c for a representation of the brain LV and additional contributing networks).

### 2.3 Neurodevelopmental alterations in the inhibitory control task architecture are linked to greater expression of genes implicated in susceptibility to stress and adversity

#### 2.3.1 Gene expression profiles linked to neurodevelopment as a function of adversity exposure and genetic risk for MDD/AD

A gene-brain PLS analysis with 10-fold cross-validation identified a gene expression LV that was validated across all test folds (*p* = 10^-5^, see Fig. 5-a, b for the spatial expression profile of the gene LV). The predicted value of the extracted gene LV showed a reliable positive correlation with the predicted value of the brain LV (cf. Fig. 4), *r* = 0.36, 95 % CI = [0.26;0.46] across all 10 test folds.

#### 2.3.2 Stress-relevant gene expression profile

Based on the brain-gene LV correlations, stress-relevant transcriptomic associations with the neurodevelopmental profile linked to adversity/genetic risk were derived from the number of stress_low genes with reliable (99.9 % CI) negative correlations with the gene LV and stress_high genes with reliable (99.9 % CI) positive correlations with the gene LV. As hypothesised, we found a positive association between our identified brain maturation profile and the gene expression patterns related to greater stress susceptibility (permutation-based *p*-value.018, see Fig. 5-c). Fig. S5 depicts the replication of these results with the left hemisphere only Schaefer atlas, as well as the left and bi-hemispheric Gordon atlas data.

As a control comparison, we verified that there was no statistically significant association between the adversity-genetic risk CCA variate and the “inverse” stress-relevant transcriptomic profile (i.e., stress_low genes with reliable (99.9 % CI) positive correlations with the gene LV and stress_high genes with reliable (99.9 % CI) negative correlations with the gene LV) (both permutation-based *p*-values >0.09). This result pattern was replicated with the Gordon atlas (both *p*s > 0.09).

### 2.4 The neurodevelopmental profile linked to adversity and genetic risk for MDD/AD is associated with areas of greater D2, Glu5R and GABA receptor density

To elucidate the overlap between the identified adversity/genetic risk-relevant brain profile and density maps of neurotransmitters which play a substantial role in psychopathology, responses to adversity and MDD/AD, more specifically, we conducted a CCA with 10-fold cross-validation. A sole mode was validated across all 10 test samples with permutation-based significance testing featuring 100,000 spatially constrained null brain maps generated with Vasa’s algorithm (*r* of. .44, *p* = 10^-5,^ Fig. 6-f). GABA, D2 and GLU5R made robust contributions to the extracted receptor density variate (Fig. 6-a, b). Specifically, the observed pattern of greater VIS and DAN functional network segregration, suggestive of accelerated maturation, overlapped areas of greater GABA receptor density (Fig. 6-e). Complementarily, the profile of poorer DMN, SM, SAL/VAN, and FPC network segregation, likely indicative of delayed neurodevelopment, was associated with regions of higher D2 and Glu5R receptor density (Fig. 6-c, d).

### 2.5 Neurodevelopmental alterations as mediators of the link between psychopathology and adversity/genetic risk for MDD/AD

Finally, we examined the relevance of the identified neurodevelopmental profile linked to - adversity and genetic risk for increasing psychopathology risk (from the two- to the three-year follow-up). To this end, we specified the moderated mediation model represented in Fig. 7. Brain maturation was indexed via patterns of functional network segregation observed during the inhibition task since these were the only scrutinised neural variables robustly associated with adversity and genetic risk in both atlases (cf. Fig. 4-a and Fig. S3-a). Given their high overlap (*r* of.62), the standardised values of the baseline and two-year follow-up adversity/genetic risk CCA variates were averaged and included as the main predictor variable. The square-root of the global psychopathology score at the three-year follow-up (residualised for the square-root of the global psychopathology score at the two-year follow-u) was the outcome variable. A dummy-coded “low/”high” “risk” variable based on a median split of the corresponding CCA variate (cf. Fig. 3), averaged across baseline and two-year follow-up, was interrogated as a moderator of the neurodevelopment-psychopathology link (see Table 1 for a comparison between the high and low vulnerability subgroups). We opted to bin the “risk” CCA variate in order to minimise multicollinearity with the continuous “risk” predictor and because we expected it to exert a categorical, rather than linear, moderating effect. The confounders listed in section “Control Variables” were introduced as covariates of no interest.

**The inhibition-relevant neurodevelopmental alterations related to adversity/genetic risk predict increases in psychopathology among the less vulnerable adolescents.** The tested moderated mediation model emerged as significant, with an index of − 0.013, SE = 0.008, 95 % CI [− 0.029; − 0.001]. Specifically, we found that increases in psychopathology risk were predicted by a neurodevelopment x adversity/genetic risk interaction, *b* = −0.188, SE = 0.066, *t*(953) = −2.852, *p* = .004. Thus, the link between the inhibition-relevant functional segregation profile and rising psychopathology risk was significant only among the less vulnerable participants, effect of.125, SE = 0.052, 95 % CI [0.022;0.227]. Accordingly, only this group showed a significant mediation of adversity/genetic risk effects on increasing psychological problems via inhibition-relevant functional brain alterations, effect of.008, SE = 0.005, 95 % CI [0.0001;0.020].

## 3 Discussion

Using longitudinal multimodal data from the ABCD study, we provide novel evidence of a psychopathology-relevant neurodevelopmental profile which is related to greater exposure to adversity and genetic susceptibility to stress in adolescence. Our work extends prior proposals that systemic inflammation mediates the negative consequences of environmental and genetic risk by derailing normative architectural brain patterns (Goldsmith et al., 2022). Specifically, we show that the functional connectomic alterations related to adversity and genetic vulnerability to MDD/AD, two disorders associated with greater stress susceptibility (Beurel et al., 2020, De Felice et al., 2022, Lutz et al., 2020), span inflammation-sensitive systems (DMN, SAL/VAN), including frontostriatal circuits implicated in motor behaviour (SM, see Fig. 4-c; cf. Goldsmith et al., 2022). We further provide evidence that the transcriptomic signature of the observed neurodevelopmental profile is enriched for genes implicated in sensitivity to stress (Nagel et al., 2020), thereby rendering it plausible that the latter may underpin the joint impact of adversity and genetic risk for MDD/AD on psychological adjustment in adolescence. Our transcriptomic results also lend support to the proposal that a predisposition towards experiencing negative mood states may constitute a correlate of AD risk in adolescence (Dafsari and Jessen, 2020, De Jager et al., 2021, Nagel et al., 2020), (partly) accounting for the alleged causal role of MDD in subsequent AD onset (cf. Harerimana et al., 2022).

Reinforcing the contribution of DA and GLU to both cognitive control and responses to adversity (DiSabato et al., 2021, Fox and Lobo, 2019, Jia et al., 2021, Qi et al., 2022, Westbrook et al., 2021, Zayed et al., 2022, Zhong et al., 2022), our observed brain profile captured the apparent delayed maturation (i.e., slower functional segregation) of the inhibition-relevant architecture, spanning areas of greater D2R and GLU5R density in functional systems that are trans-diagnostically implicated in psychopathology (DMN, SM, SAL/VAN, Control, Javaheripour et al., 2021; Kebets et al., 2019; McTeague et al., 2017). As such, our results extend previous rodent reports of GLU5R involvement in stress responses and susceptibility to psychological dysfunction following adverse experiences (Joffe et al., 2022, Kim et al., 2022). They further reaffirm the well-documented relevance of D2R to psychopathology, particularly psychosis (Dandash et al., 2017), and effortful cognitive control processes (Salami et al., 2018, Salami et al., 2019).

Complementarily, we provided evidence linking adversity and genetic risk for MDD/AD (APOE-unrelated) to accelerated development (i.e., faster functional segregation) of visual and goal-directed attentional (VIS/DAN) processing networks. These systems have been implicated in MDD pathology (e.g., Ray et al., 2021; Xue et al., 2022) and symptom amelioration following treatment (Moreno-Ortega et al., 2019, Petrican et al., 2019). They have been further shown to be susceptible to degeneration among individuals with greater no-APOE-based risk for AD (cf. Frisoni et al., 2022). At the molecular level, the faster maturing VIS/DAN areas overlapped regions of greater GABA receptor density, thereby indirectly testifying to the foundational role of GABA-ergic balance in functional network development (Kalemaki et al., 2021, Weitz et al., 2019).

Interestingly, we found suggestive evidence that the severity of environmental and genetic risk factors moderates the psychological impact of their associated neurodevelopmental alterations. Specifically, the observed pattern of accelerated VIS/DAN, but delayed SM and association system maturation/functional segregation was more strongly associated with subsequent increases in psychopathology among the less vulnerable adolescents. Indeed, it was only in this group that we detected a robust link between the adversity/genetic risk-related neurodevelopmental profile and growing psychological dysfunction, whereas a relationship in the opposite direction tended to emerge in the more vulnerable group. Our findings imply that typically maladaptive neurodevelopmental alterations may nonetheless foster adjustment among adolescents who are exposed to greater adversity and are genetically susceptible to stress. They further raise the possibility that the functional relevance of the observed delayed vs accelerated neurodevelopmental patterns may vary between the low and high vulnerability groups. Specifically, among the less vulnerable participants, greater risk for psychopathology likely stemmed from a lower self-regulatory capacity, which in turn was linked to delayed maturation of association systems that underlie cognitively effortful responses to adversity (Koban et al., 2021, McTeague et al., 2017). The negative consequences of accelerated maturation for the goal-directed attentional system are thought to emerge over longer timescales (Tooley et al., 2021) and thus are less likely to have contributed to the effects observed in our low vulnerability group. Complementarily, the trending protective effect of neurodevelopment observed among the more vulnerable youths fits well with the stress acceleration hypothesis (Callaghan and Tottenham, 2016), as it plausibly reflects the immediate self-regulatory benefits associated with the faster maturation of attentional control relevant circuits (i.e., DAN). Nonetheless, more in-depth investigation of the brain maturation mechanisms that promote adaptation among more vulnerable youths, and their long-term consequences (cf. McLaughlin et al., 2014, 2016), would certainly be worth undertaking in the future.

Our present research underscores the importance of conducting more targeted, neurotransmitter system-specific investigations into the relationships among genetic susceptibility to stress-related conditions, including MDD/AD, resilience versus vulnerability to psychopathology and incentive processing. For instance, distinguishable GLU neuron populations in the ventral tegmental area (VTA) have been implicated in coding rewards and losses (McGovern et al., 2021), while GABA and GLU neurons in the basal ganglia have been shown to code for rewards and threats, respectively (Al-Hasani et al., 2021, Stephenson-Jones et al., 2020). Extending these findings, it would be establishing whether the observed GLU/GABA link to adversity/genetic risk for MDD/AD may increase vulnerability to psychological dysfunction through distinguishable pathways (e.g., threat/loss- vs reward-linked). Such a line of enquiry would be well-served by studies targeting neurotransmitter-specific substrates of whole-brain functional network interactions, while accounting for age-related modulation (Ferrara et al., 2021), including effective connectivity patterns between subcortical and cortical regions. This work would further benefit from experimental manipulations targeting the variety of mental processes (e.g., positive emotion upregulation vs negative emotion downregulation, Min et al., 2022) and cells-to-networks neurobiological mechanisms underlying psychopathology risk as a function of stressor and genetic vulnerability.

Broadly, our present findings call for more fine-grained investigations of candidate neurobiological mechanisms underpinning the inter-relationships among adversity, stress susceptibility and genetic vulnerability for MDD and AD, respectively. Inflammatory processes linked to hypothalamic-pituitary-adrenal (HPA) axis dysregulation (Dafsari and Jessen, 2020) would warrant special attention, particularly zooming in on alterations observed in astrocytes, microglia and mitochondria, given their susceptibility to stress, glutamatergic/GABA-ergic modulation, as well as role in neurodegeneration and psychological resilience (Bellenguez et al., 2022, Brivio et al., 2022, Cathomas et al., 2022, Cheng et al., 2022, Jiwaji et al., 2022, Mederos et al., 2021, Miller et al., 2022; Snijders et al., 2021; Wightman et al., 2021). Additionally, a better understanding of how adversity may impact the interaction between the HPA and hypothalamic-pituitary-gonadal (HPG) axes could provide key insights into how pubertal development may modulate the emergence of short- versus long-term (mal)adaptive responses to adverse rearing environments (Colich and McLaughlin, 2022). Such a line of investigation would be certainly warranted since recent evidence points to the unique role of gonadal hormons in optimising cognitive control performance (Ravindranath et al., 2022), thereby indirectly impacting the capacity to withstand stress.

## 4 Limitations and future directions

The limitations of our present research open several new avenues of enquiry. First, the ABCD study uses traditional laboratory tasks, which, in comparison with naturalistic paradigms, tend to be less accurate in mimicking the neurocognitive patterns evoked in real-world settings (Gal et al., 2022, Jovanovic et al., 2022). Consequently, replication and extension of our findings in studies featuring a movie watching paradigm, for instance, could yield further insights into the results herein documented. Second, we only assessed the relevance of functional neurodevelopment to psychopathology risk. However, patterns of functional network interactions underpinning optimal adjustment have been shown to vary by domain (i.e., cognition, personality, mental health) (Chen et al., 2022). As such, key insights could be gained by further probing our present findings on psychopathology risk through investigations on the neurodevelopmental mechanisms underpinning the interactive effect of adversity and genetic risk for MDD/AD on profiles of cognition and/or personality.

Third, our investigation focused on the link between genetic risk factors and functional brain maturation. Stronger genetic effects are though reportedly observed on structural (relative to functional) brain indices, while structure-function coupling in connectomic features is also under substantial genetic modulation (Arnatkeviciute et al., 2021a, Arnatkeviciute et al., 2021b, Baum et al., 2020, Gu et al., 2021). Consequently, investigations of synchronised structure-function development would augment the findings herein reported, particularly given the robust predictive power of both white and grey matter indices for AD and MDD (Huang et al., 2022, Lu et al., 2022), as well as psychopathology risk (Aggarwal et al., 2022, Fenchel et al., 2022, Lalousis et al., 2022, Park et al., 2022, Patel et al., 2022, Zhao et al., 2021a, Zhao et al., 2021b).

Fourth, our analyses only provided suggestive evidence on the chemoarchitectural correlates of adversity/genetic-risk-related neurodevelopmental alterations. A finer grained dynamic representation of the underlying multiscale interactions could though be painted through further cross-species investigations probing specific receptor types (e.g., D1 vs D2, Simpson et al., 2022), as well as interactions among multiple neurotransmitter systems (cf. Mei et al., 2022) within an experimental paradigm. Fifth, concerns regarding model complexity and replicability drove our decision to control for, rather than probe, sex differences in any of our observed effects. However, there are reliable sex differences in stress responses (Caradonna et al., 2022, Kuhn et al., 2022), as well as in the genetic architecture underlying complex traits (Bernabeu et al., 2021). These highlight the need for cross-species comparative research to characterise sex-specific neurogenetic substrates of psychopathology, including joint and unique compensatory mechanisms linked to adversity exposure and AD/MDD vulnerability, respectively.

Sixth, pubertal status was not included in our analyses because investigation of its putative role in partly mediating the impact adversity exposure on neurodevelopment (Eck and Bangasser, 2020, Laube et al., 2020) was beyond the scope of our present study. Nonetheless, targeted investigations into the role of pubertal hormones in mediating environmental adversity and genetic risk effects on brain maturation pace via distinct neurotransmitter systems would be certainly worth pursuing in the future.

Seventh, while our present study focused on the impact of environmental and genetic risk variables, there is a strong need for further investigation of potential protective factors. Of special interest are the pathways through which lifestyle choices (e.g., aerobic exercise, De Miguel et al., 2021; Ludyga et al., 2022; diet, Ratsika et al., 2021) and social relationships, including parental and peer presence in childhood/adolescence (Rogers et al., 2022; Willoughby et al., 2021) protect against psychological problems. In particular, cross-species investigations of neuroplasticity-mediated increases in resilience to psychopathology (Nosjean and Granon, 2021) may be pivotal to designing interventions in adolescence and young adulthood to decelerate or annihilate progression towards dementia in older age.

## 5 Conclusions

Exposure and genetic susceptibility to stress were related to distinguishable patterns of accelerated (VIS/DAN, GABA-linked) and delayed (SM/association systems, D2R/GLU5R-linked) functional network development, which were differentially predictive of subsequent psychopathology risk among the more vs less vulnerable adolescents. Promising support emerged for the proposed role of broad negative emotionality as an earlier life precursor to AD, likely to account for the alleged causal impact of MDD on dementia onset in older adulthood.

## Data statement

The raw data are available at https://nda.nih.gov/abcd upon completion of the relevant data use agreements. The ABCD data repository grows and changes over time. The ABCD data used in this report came from Adolescent Brain Cognitive Development Study (ABCD) - Annual Release 4.0 #1299. DOIs can be found at http://dx.doi.org/10.15154/1523041.

## Code availability

We used already existing code, as specified in the main text with links for free download.

## Declaration of Competing Interest

The authors declare that they have no known competing financial interests or personal relationships that could have appeared to influence the work reported in this paper.

## Data Availability

Data will be made available on request.
