# Supplementary material for: Adolescent neurodevelopment and psychopathology: The interplay between adversity exposure and genetic risk for accelerated brain ageing
Source: Dev Cogn Neurosci. 2023 Mar 15;60:101229. doi: 10.1016/j.dcn.2023.101229 (PMC10041470; doi:10.1016/j.dcn.2023.101229)
Supplement: Supplementary file 1 — Supplementary material [file mmc1.docx]

Table S1

*Demographic, Adversity and Genetic Risk Information for Participants with Available Data on All Measures on Interest*

**
