# Supplementary figures and images for: Adolescent neurodevelopment and psychopathology: The interplay between adversity exposure and genetic risk for accelerated brain ageing
Source: Dev Cogn Neurosci. 2023 Mar 15;60:101229. doi: 10.1016/j.dcn.2023.101229 (PMC10041470; doi:10.1016/j.dcn.2023.101229)

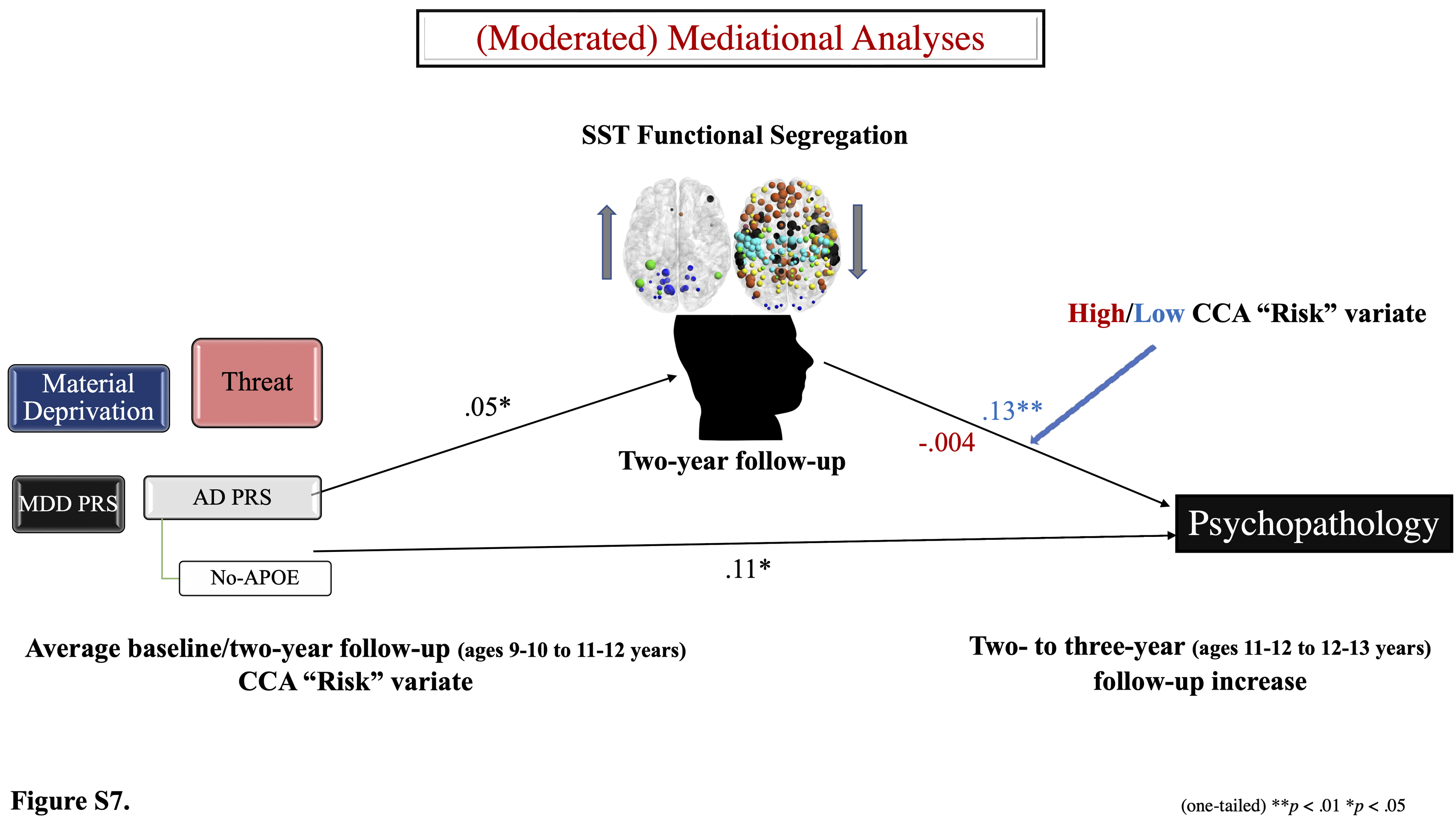

Supplement: Supplementary file 2 — Supplementary material [file mmc10.jpg]

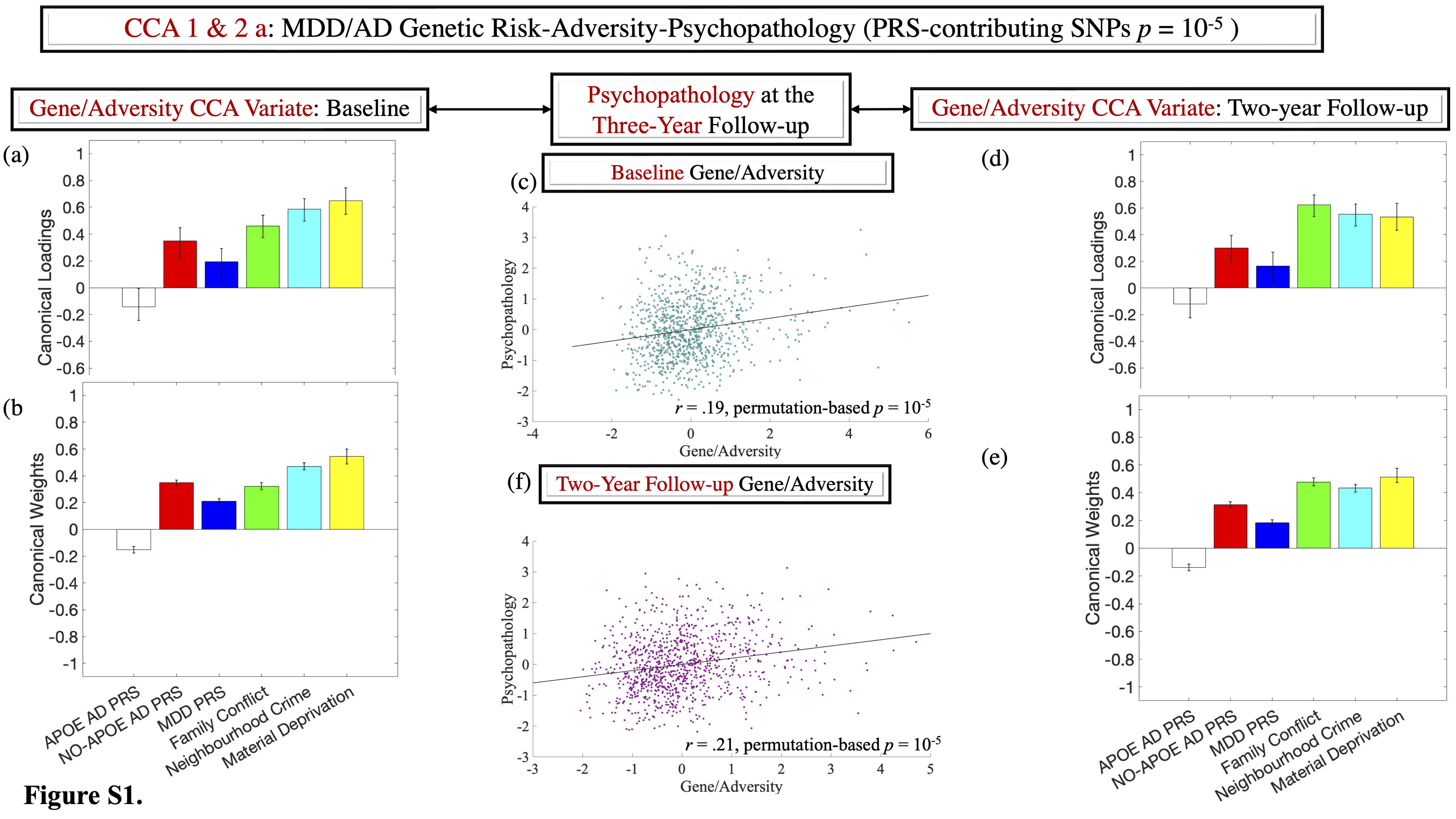

Supplement: Supplementary file 5 — Supplementary material [file mmc4.jpg]

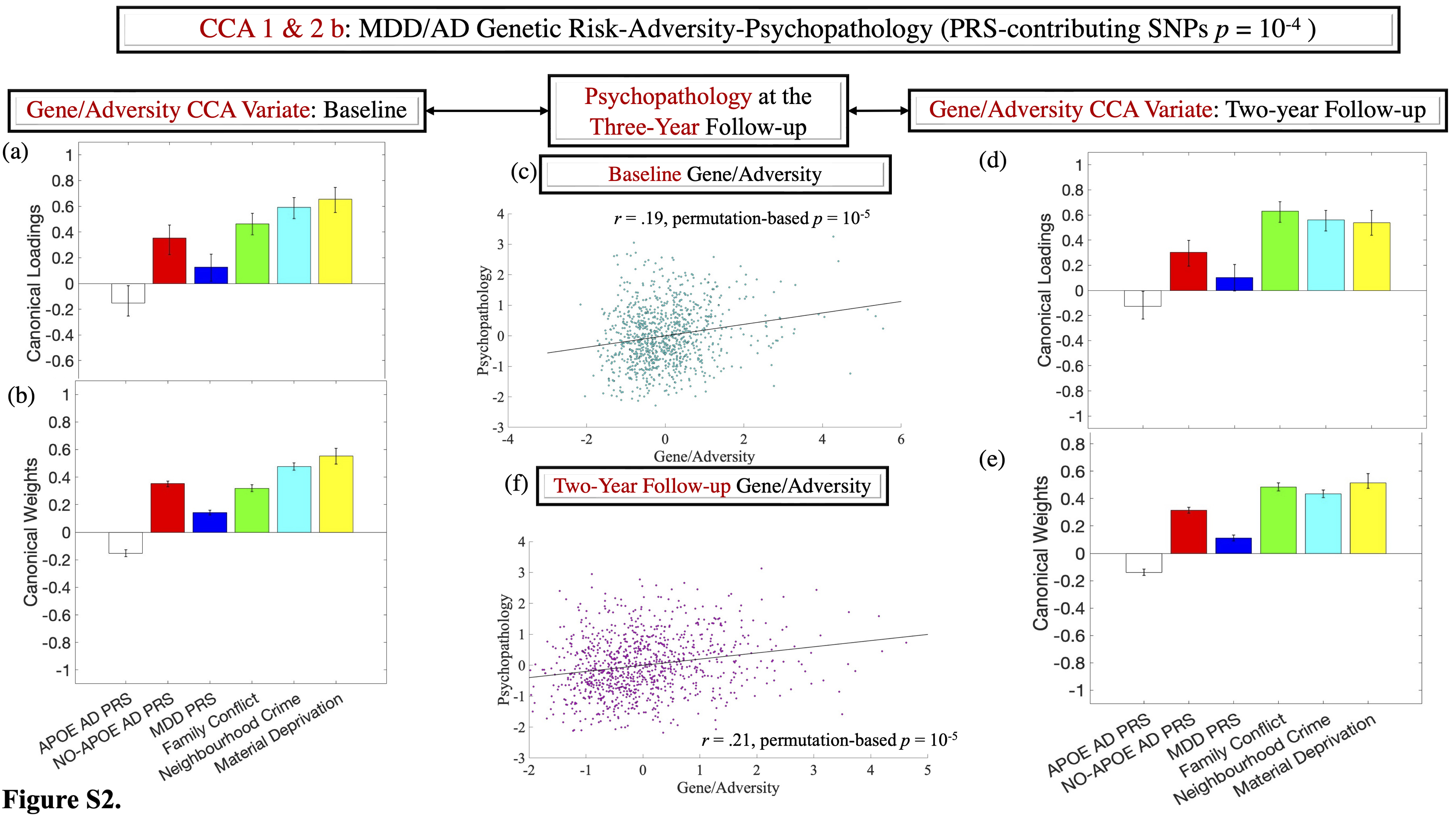

Supplement: Supplementary file 6 — Supplementary material [file mmc5.jpg]

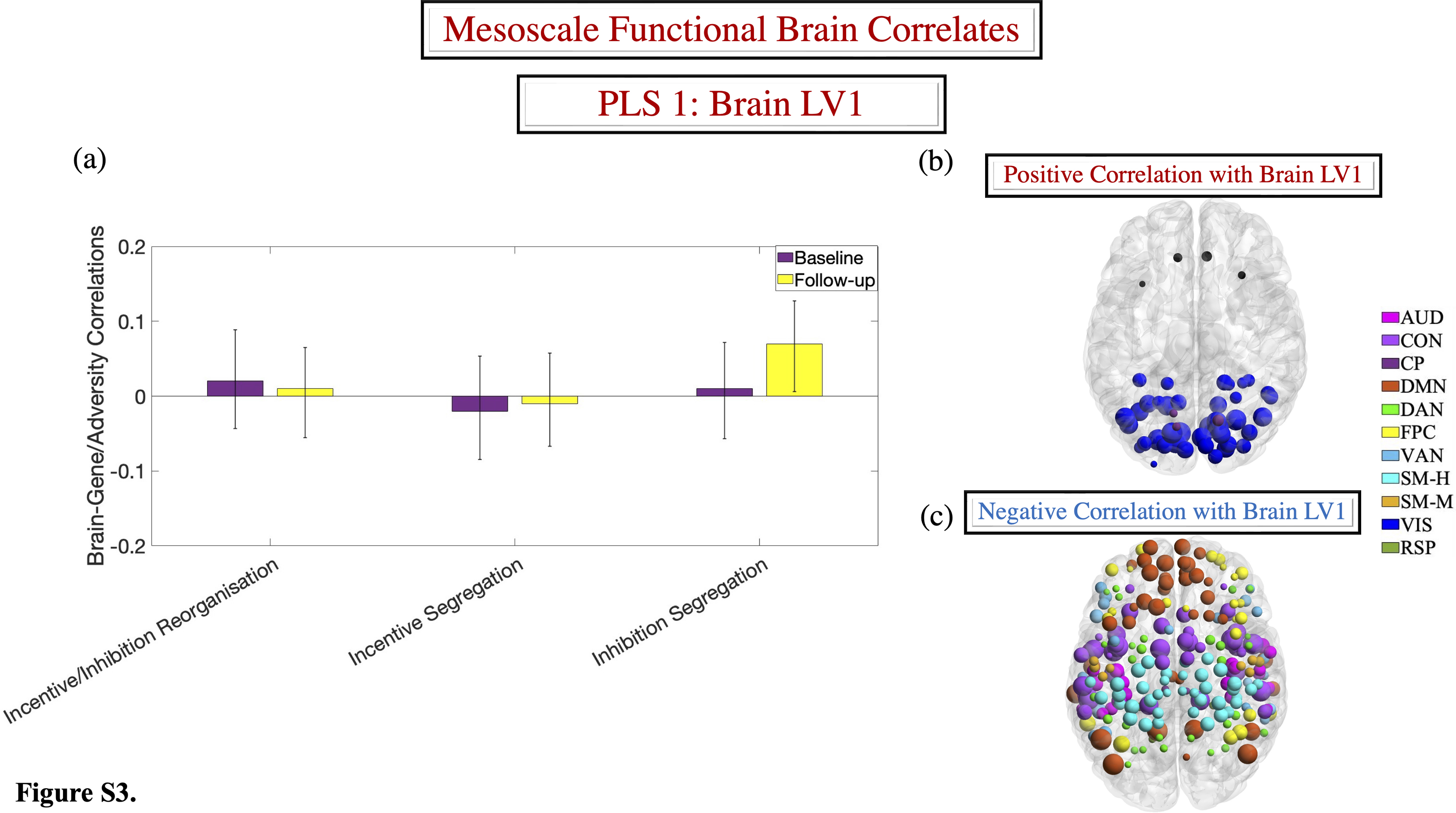

Supplement: Supplementary file 7 — Supplementary material [file mmc6.jpg]

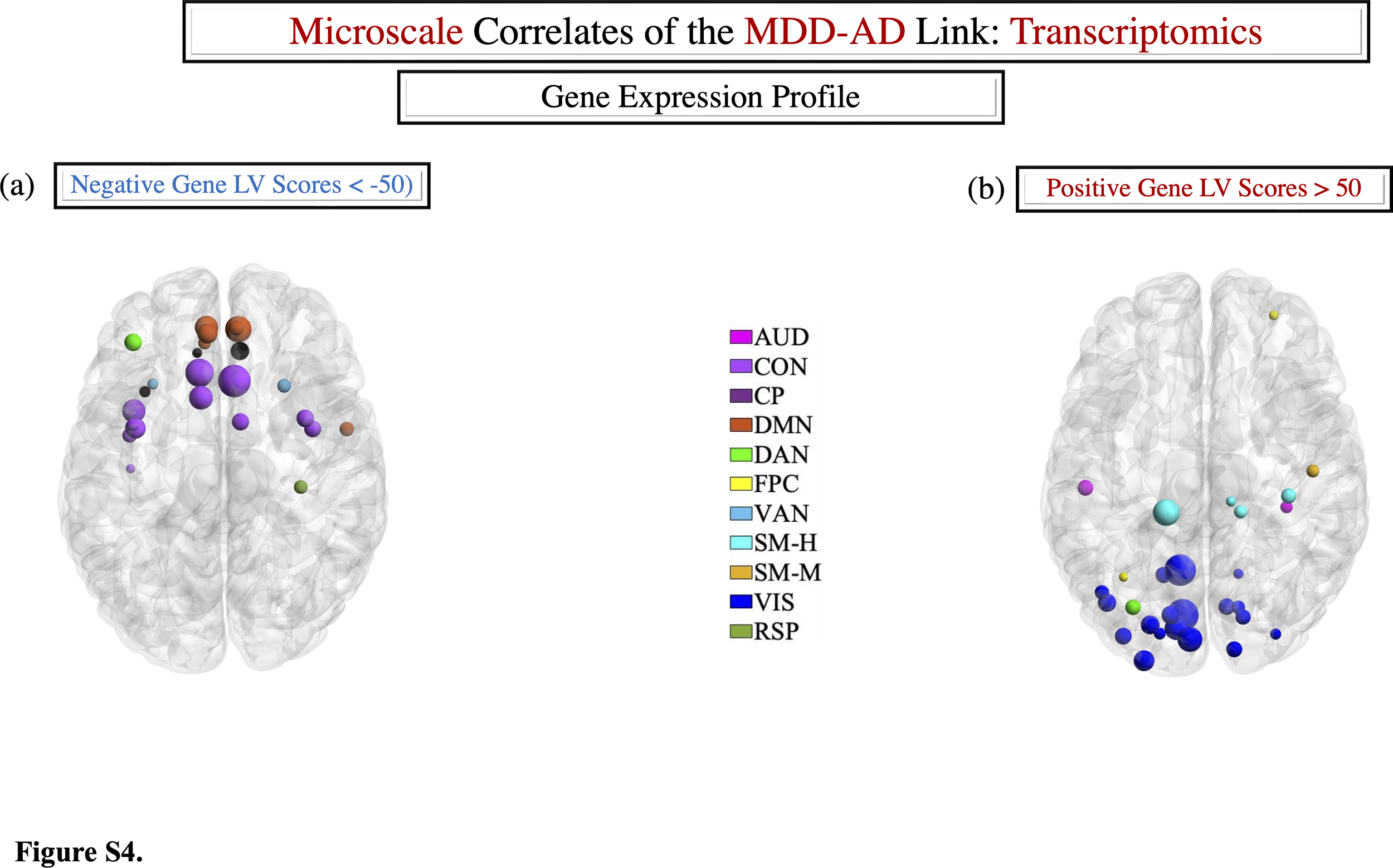

Supplement: Supplementary file 8 — Supplementary material [file mmc7.jpg]

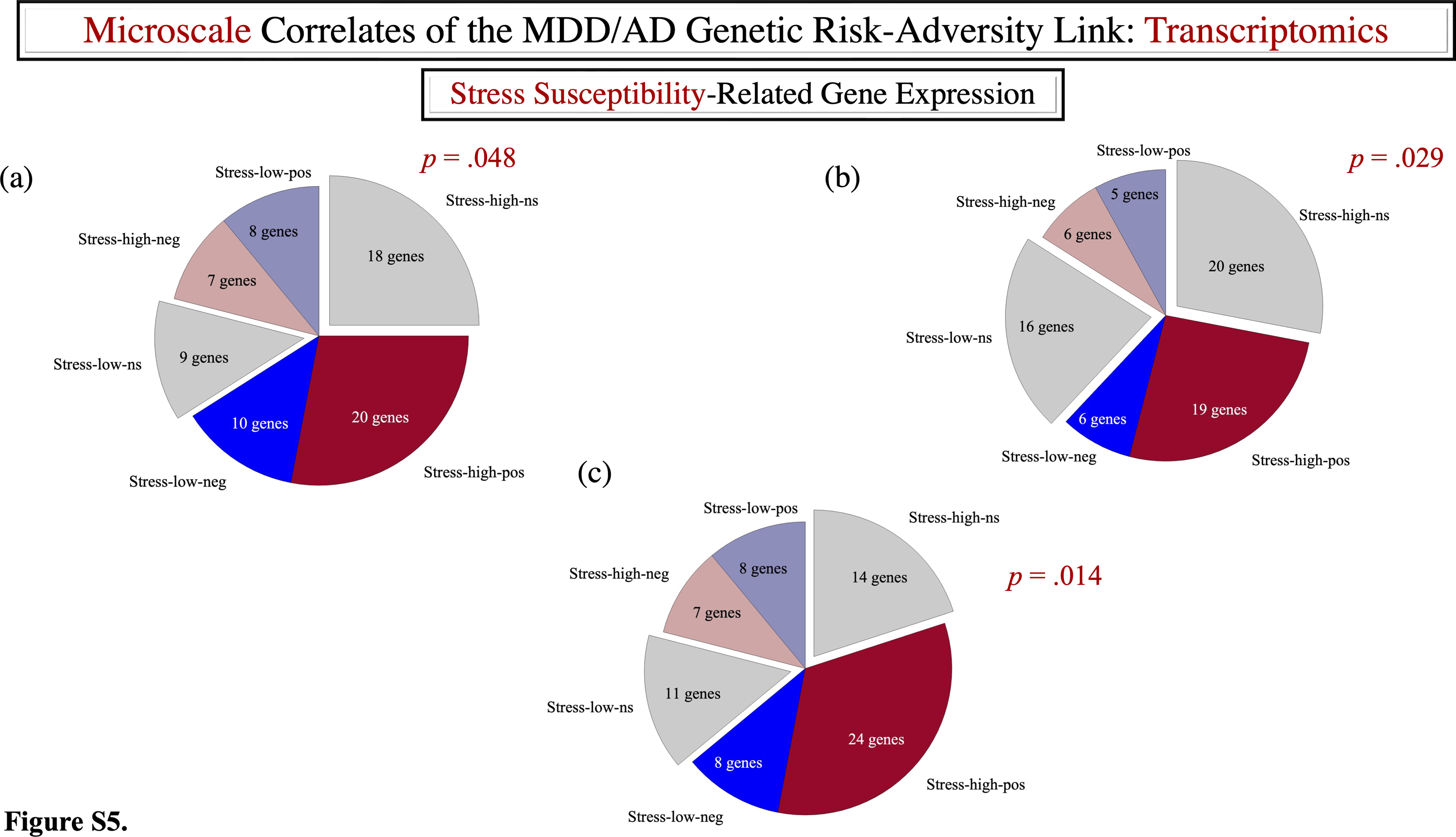

Supplement: Supplementary file 9 — Supplementary material [file mmc8.jpg]

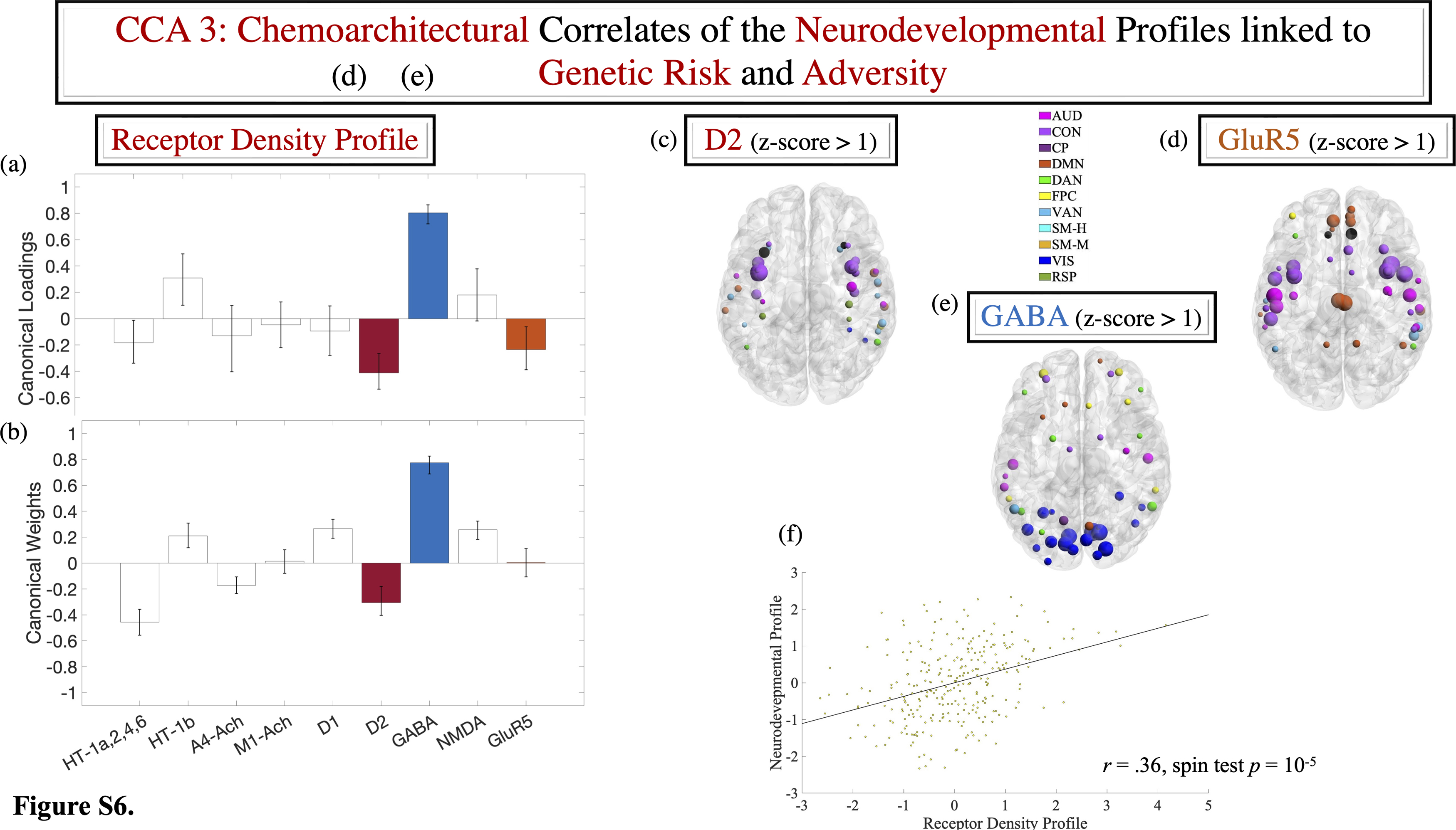

Supplement: Supplementary file 10 — Supplementary material [file mmc9.jpg]
