# Supplementary material for: Adolescent neurodevelopment and psychopathology: The interplay between adversity exposure and genetic risk for accelerated brain ageing
Source: Dev Cogn Neurosci. 2023 Mar 15;60:101229. doi: 10.1016/j.dcn.2023.101229 (PMC10041470; doi:10.1016/j.dcn.2023.101229)
Supplement: Supplementary file 3 — Supplementary material [file mmc2.docx]

Table S2

*MNI Coordinates, Anatomical Location and Functional Network Allegiance for the Schaefer and Gordon ROIs with No Reliable Gene Expression Data*

|  | MNI Coordinates | | | | Anatomical Location | Functional Network Allegiance |
| --- | --- | --- | --- | --- | --- | --- |
| Schaefer atlas | X | Y | | Z |  |  |
| 1 | -50 | | -12 | 14 | Primary sensory cortex | SM- B |
| 2 | -50 | | -16 | 44 | Primary sensory cortex | SM-B |
| 3 | -12 | | -4 | 72 | BA6 | SAL-VAN |
| Gordon atlas |  | |  |  |  |  |
| 1 | -27.5 | | -37.2 | 61.4 | Sensory association cortex | SM-H |
| 2 | -44.8 | | -54 | 14.6 | Angular gyrus | VAN |
| 3 | -46.3 | | -41.4 | 25.9 | Angular gyrus | AUD |
| 4 | -45.4 | | 28.8 | 0.8 | BA45 | VAN |
| 5 | -52.2 | | -14.1 | 15.2 | Primary sensory cortex | AUD |
| 6 | -34.7 | | 5.6 | 34 | Frontal eye fields | DAN |
| 7 | -41.6 | | 8.7 | 22.2 | BA44 | DAN |
| 8 | -27.5 | | 53.6 | 0 | BA10 | DMN |
| 9 | -26.6 | | 46.8 | 20.9 | BA10 | CON |
| 10 | -35.7 | | 33.1 | 32 | BA9 | DAN |
| 11 | -38.7 | | 4.8 | 48.4 | BA6 | VAN |
| 12 | 6.7 | | 5 | 55.9 | BA6 | CON |
| 13 | 16.2 | | 0.8 | 67.5 | BA6 | CON |
| 14 | 28 | | -34.8 | 63.1 | Primary sensory cortex | SM-H |
| 15 | 30.6 | | 22.8 | -4.7 | Insula | SAL |
| 16 | 26.8 | | -55 | 54.2 | Visuomotor cortex | VIS |
| 17 | 7.7 | | -85.6 | 31.6 | Visual association cortex | VIS |
| 18 | 54.2 | | -13.6 | 16.9 | Supramarginal gyrus | AUD |
| 19 | 31.2 | | -45.6 | -5.8 | Visual association cortex | VIS |
| 20 | 20.4 | | -87.3 | -6.6 | Secondary visual cortex | VIS |

Note. BA = Brodmann area. Schaefer networks: SAL-VAN = salience/ventral attention. SM-B =somatomotor-B. Gordon networks: AUD = auditory. CON = cingulo-opercular. DMN = default mode. DAN = dorsal attention. VAN = ventral attention. SM-H = somatomotor-hand. VIS = visual.
