# Supplementary material for: Adolescent neurodevelopment and psychopathology: The interplay between adversity exposure and genetic risk for accelerated brain ageing
Source: Dev Cogn Neurosci. 2023 Mar 15;60:101229. doi: 10.1016/j.dcn.2023.101229 (PMC10041470; doi:10.1016/j.dcn.2023.101229)
Supplement: Supplementary file 4 — Supplementary material [file mmc3.docx]

**Supplementary Figure Captions**

*Figure S1.* Correlation [panels a, d] and standardised coefficients [panels b, e] describing the relationship between the observed adversity/genetic risk variables and the predicted value of their corresponding canonical variate across all test CCAs. The PRS-contributing SNPs had an associated *p*-value < 10^-5^ Panels (c) and (f) contain the scatter plot describing the linear relationship between the predicted value of the two variates at baseline and the two-year follow-up, respectively. Error bars are based on the bootstrapping procedure (99.9% confidence intervals) as described in the text. PRS = polygenic risk score. SNP = single nucleotide polymorphism. CCA = canonical correlation analysis.

*Figure S2.* Correlation [panels a, d] and standardised coefficients [panels b, e] describing the relationship between the observed adversity /genetic risk variables and the predicted value of their corresponding canonical variate across all test CCAs. The PRS-contributing SNPs had an associated *p*-value < 10^-4^ Panels (c) and (f) contain the scatter plot describing the linear relationship between the predicted value of the two variates at baseline and the two-year follow-up, respectively. Error bars are based on the bootstrapping procedure (99.9% confidence intervals) as described in the text. PRS = polygenic risk score. SNP = single nucleotide polymorphism. CCA = canonical correlation analysis.

*Figure S3*. The brain LV from the behavioral-PLS analysis linking the adversity/genetic risk CCA variate to functional brain development. The PLS-extracted profile, in which the gene/adversity scores accounted for 50% of the variance in the brain data, was validated across all 10 test folds (*p* = .022). Panel (a) shows the correlations between the CCA variate and the predicted brain scores in each condition (based on the 10-fold cross-validation procedure described in the main text in reference to the Schaefer atlas). Panels (b) and (c) depict the Gordon ROIs with robust loadings (based on cross-validated 99.9% confidence intervals, as described in the main text) on the LV in panel (a) and visualized with the BrainNet Viewer (http://www.nitrc.org/projects/bnv/). ROI colours reflect Gordon et al.’s network assignments. In panels (b) and (c), the size of the ROIs is proportional to their strength of their correlation with the brain LV. In panel (a), error bars are the 95% confidence intervals from the bootstrap procedure. Confidence intervals that do not include zero reflect robust correlations between the adversity/genetic risk CCA variate and the predicted value of the brain score in a given condition across all participants. CCA = canonical correlation analysis. LV= latent variable. Gordon networks: AUD = auditory; CON = cingulo-opercular; CP = cingulo-parietal; DMN = default mode; DAN = dorsal attention; FPC = frontoparietal; RSP = retrosplenial/temporal; SM-H =somatomotor-hand; SM-mouth = somatomotor mouth; SAL = salience; VAN = ventral attention; VIS =visual.

*Figure S4*. Results of the PLS gene expression analyses based on the Gordon atlas. Panels (a) and (b) represent the spatial expression map of the gene LV identified in the bi-hemispheric gene-brain behavioral PLS analysis. As reported for the Schaefer atlas data, the gene LV extracted from both the left-hemisphere and bi-hemispheric data was validated across all 10 test folds (permutation-based *p*s of 2 x 10^-4^ and 10^-5^ , respectively). The predicted value of the gene LV was positively correlated with the predicted value of the brain LV (cf. Figure S3), *r* = .32, 95% CI = [.16; .47] (left-hemisphere-only data) and *r* = .31, 95% CI = [.20; .41] (bi-hemispheric data) across all testing folds. For presentational purposes only, panels (a) and (b) depict only the ROIs with predicted gene LV scores greater than 50 in absolute value (the predicted LV scores had been centered around 0). The ROIs were visualized with the BrainNet Viewer (http://www.nitrc.org/projects/bnv/). ROI colours reflect Gordon et al.’s network assignments and their size is proportional to how strongly they express the gene LV (i.e., the absolute value of the associated predicted brain score, as derived from the 10-fold cross-validation procedure). CCA = canonical correlation analysis. LV= latent variable. Gordon networks: AUD = auditory; CON = cingulo-opercular; CP = cingulo-parietal; DMN = default mode; DAN = dorsal attention; FPC = frontoparietal; RSP = retrosplenial/temporal; SM-H =somatomotor-hand; SM-mouth = somatomotor mouth; SAL = salience; VAN = ventral attention; VIS =visual.

*Figure S5*. Results of the stress susceptibility overlap analyses, using only left-hemisphere Schaefer (a) Gordon (b) or bi-hemispheric Gordon (c) atlas data. The results described in panel (a) are based on a PLS analysis using only left-hemisphere data from the Schaefer atlas.

This gene-brain PLS analysis with 10-fold cross-validation identified a gene expression LV that was validated across all test folds (*p* = 2 x 10^-5^). The predicted value of the extracted gene LV was positively correlated with the predicted value of the brain LV (cf. Figure 4), *r* = .37, 95% CI = [.21; .51] across all 10 test folds. The results described in panels (b) and (c) are based on the gene PLS analyses described in the legend for Figure S4.

*Figure S6*. Receptor density maps linked by CCA to the adversity/genetic risk-relevant neurodevelopmental profile (cf Figure S3). Correlation [panel a] and standardised coefficients [panel b] describing the relationship between the observed receptor density maps and the predicted value of their corresponding canonical variate across all test CCAs. Panels (c)-(e) depict the D2, Glu5R and GABA receptor density maps thresholded at a z-score value > 1 for presentational purposes only. Panel (f) contains the scatter plot describing the linear relationship between the predicted value of the brain LV extracted with PLS (cf. Figure S3) and the predicted value of the receptor density CCA variate. The ROIs were visualized with the BrainNet Viewer (http://www.nitrc.org/projects/bnv/). ROI colours reflect Gordon et al.’s network assignments and their size is proportional to the density of the respective receptor. CCA = canonical correlation analysis. GLU = glutamate. GABA = gamma-aminobutyric acid. D = dopamine. AUD = auditory; CON = cingulo-opercular; CP = cingulo-parietal; DMN = default mode; DAN = dorsal attention; FPC = frontoparietal; RSP = retrosplenial/temporal; SM-H =somatomotor-hand; SM-mouth = somatomotor mouth; SAL = salience; VAN = ventral attention; VIS =visual.

*Figure S7*. (Moderated) mediational models linking the adversity/genetic risk CCA variate to increases in psychopathology from the two- to the three-year follow-up. These analyses used a one-tailed *p*-value of .05 and 90% confidence intervals, which were deemed appropriate for replication purposes. In line with the results obtained with Schaefer atlas data, we observed a robust moderated mediation effect, with an index of -.007, SE = .006, 90% CI [-.017; .000], driven by a significant neurodevelopment x ELA/genetic risk interaction, *b* = -.136, SE = .067, *t*(953) = -2.009, one tailed *p* = .022. Thus, as with the Schaefer atlas data, the link between the inhibition-relevant functional segregation profile and rising psychopathology risk was significant only among the less vulnerable participants, effect of .132, SE = .050, 90% CI [.049; .214]. Accordingly, only this group showed a marginally significant mediation of ELA/genetic risk effects on increasing psychological problems via inhibition-relevant functional brain alterations, effect of .007, SE = .005, 95% CI [.000; .015]. CCA = canonical correlation analysis.
